# Deep learning enables fast, gentle STED microscopy

**DOI:** 10.1038/s42003-023-05054-z

**Published:** 2023-06-27

**Authors:** Vahid Ebrahimi, Till Stephan, Jiah Kim, Pablo Carravilla, Christian Eggeling, Stefan Jakobs, Kyu Young Han

**Affiliations:** 1 CREOL, The College of Optics and Photonics, University of Central Florida, Orlando, FL USA; 2 Department of NanoBiophotonics, Max Planck Institute for Multidisciplinary Sciences, Göttingen, Germany; 3 Department of Neurology, University Medical Center Göttingen, Göttingen, Germany; 4 Department of Cell and Developmental Biology, University of Illinois at Urbana-Champaign, Urbana, IL USA; 5 Leibniz Institute of Photonic Technology e.V., Jena, Germany, member of the Leibniz Centre for Photonics in Infection Research (LPI), Jena, Germany; 6 Faculty of Physics and Astronomy, Institute of Applied Optics and Biophysics, Friedrich Schiller University Jena, Jena, Germany; 7 Jena School for Microbial Communication, Friedrich Schiller University Jena, Jena, Germany; 8 Medical Research Council Human Immunology Unit, Weatherall Institute of Molecular Medicine, University of Oxford, Oxford, UK; 9 Translational Neuroinflammation and Automated Microscopy, Fraunhofer Institute for Translational Medicine and Pharmacology ITMP, Göttingen, Germany

**Keywords:** Fluorescence imaging, Machine learning

## Abstract

STED microscopy is widely used to image subcellular structures with super-resolution. Here, we report that restoring STED images with deep learning can mitigate photobleaching and photodamage by reducing the pixel dwell time by one or two orders of magnitude. Our method allows for efficient and robust restoration of noisy 2D and 3D STED images with multiple targets and facilitates long-term imaging of mitochondrial dynamics.

## Abstract (web summary)

Deep learning enables restoration of noisy 2D and 3D STED images for long-term super-resolution imaging.

## Introduction

Stimulated emission depletion microscopy (STED)^1,2^ is a super-resolution fluorescence imaging technique that can reveal biological structures in live cells with greater than 50 nm resolution^3^. Hereby, the effective fluorescence area is confined to nanoscales by overlapping the diffraction-limited excitation spot with a fluorescence-depleting spot exhibiting a central intensity of zero (such as a doughnut-shaped spot). Increasing the intensity of the depletion beam leads to an increase in resolution but often causes adverse effects such as photobleaching^4^ and phototoxicity^5^, preventing long-term monitoring of samples. Although optimized optical parameters^2^, multiple off states^6^, exchangeable fluorophores^7,8^, or sophisticated illumination^9^ and data acquisition schemes^10–12^ can circumvent these problems to some extent, the improvement is often small, or the choice of fluorophores is limited. In principle, reducing the STED exposure time can decrease photodamage^5^; however, a short pixel dwell time results in a poor signal-to-noise ratio (SNR) and consequently degrades image resolution^13^.

Emerging deep learning approaches have proposed different solutions to address the tradeoffs between spatial/temporal resolution, SNR, and phototoxicity^14–16^. In particular, converting confocal images to high-resolution STED images called cross-modality image restoration has shown promising results^17,18^. Here, we show that denoising STED images for image restoration is advantageous over other deep learning methods and can significantly enhance the performance of STED microscopy, i.e., the increased imaging speed and the extended observation time.

## Results and discussion

**Fig. 1:**
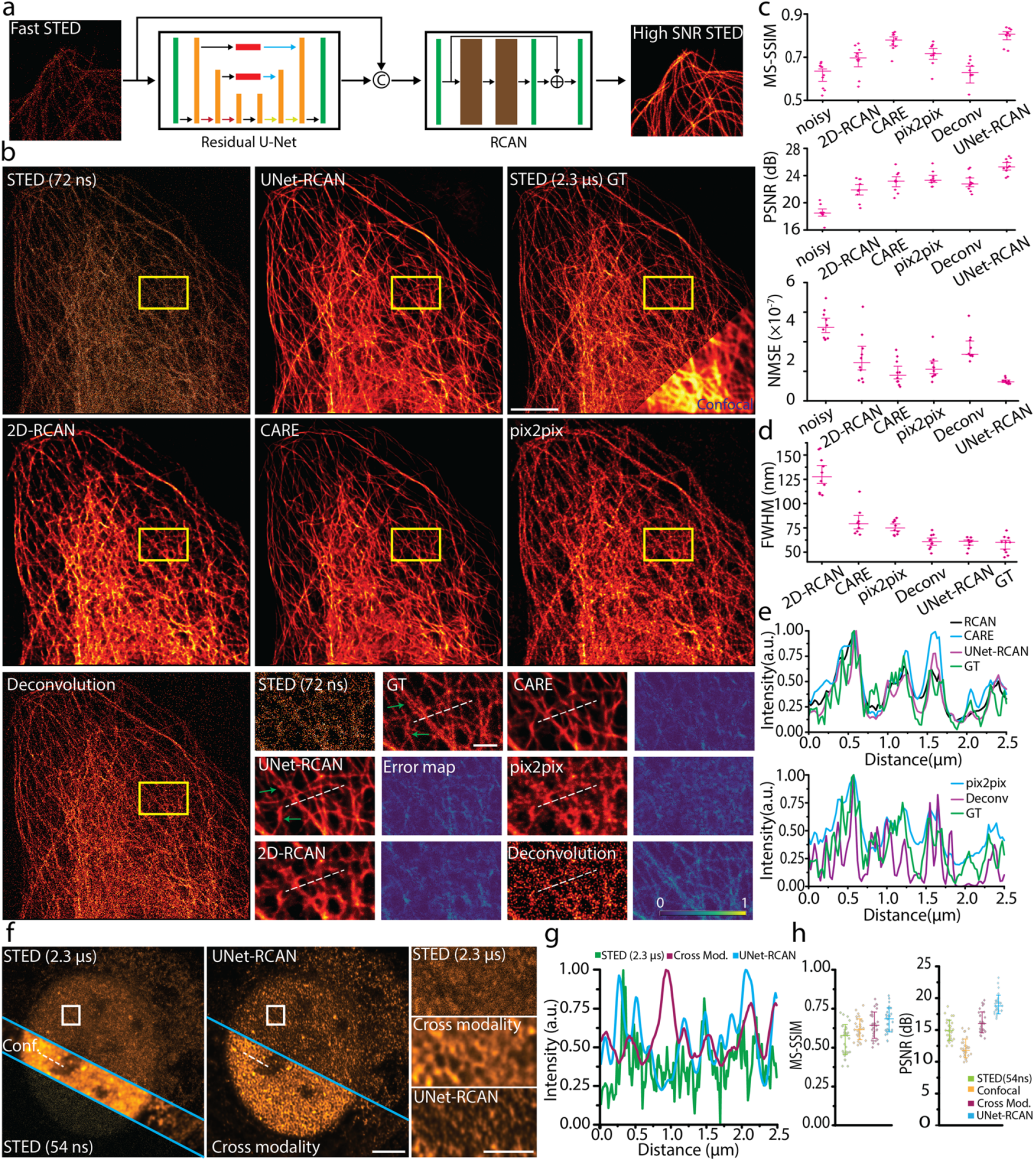
Restoration of noisy STED images by UNet-RCAN. **a** The network architecture of two-step image restoration. **b** Restoration results of UNet-RCAN, 2D-RCAN, CARE, pix2pix, and deconvolution on noisy 2D-STED images (*Δt* = 72 ns) for β-tubulin (STAR635P) in U2OS cells in comparison to the ground-truth STED data (GT; *Δt* = 2.3 μs). Error maps and arrows show the deviations of prediction results from the ground-truth. **c** Quantitative comparisons of the predicted results with ground-truth STED images (GT; *Δt* = 2.3 µs) for the methods used in **b**. Mean and standard deviation are displayed (*n* = 10). **d** Resolution analysis by measuring full width at half maximum of line profiles for the predicted results. Mean and standard error of mean are displayed (*n* = 10). **e** Line profiles along the white dashed lines in **b**. **f** Comparison of cross-modality and denoising methods for restoring high SNR STED images. Cross-modality used confocal images as input. Histone markers (H3K9ac) were labeled with Atto647N in U2OS cells. **g** Line profiles along the white dashed lines in **f**. **h** Quantitative metrics of the predicted results by cross-modality and denoising (STED = 54 ns). Scale bars, 5 μm **b**, **f** and 1 µm for their magnified regions.

We used a two-step prediction architecture based on a U-Net^19^, and a residual channel attention network (RCAN)^20^ (UNet-RCAN), in which a single U-Net restores the broad contextual information and an RCAN reconstructs the final super-resolution images (Fig. 1a, Supplementary Fig. 1). For training and testing, we acquired multiple pairs of low SNR STED images at a short pixel time (*Δt*) and high SNR STED images at a long pixel time. If necessary, a drift correction was applied for the registration of each pair (Supplementary Fig. 2). Alternatively, we obtained high SNR images and generated low SNR counterparts by adding noise (Methods), which corresponded well with the results of the sequentially acquired training set (Supplementary Fig. 3). We generally experienced that 20 image data sets were enough to train our image restoration algorithm.

To investigate the performance of our network on restoring high SNR STED microscopy data, we obtained 2D-STED images of microtubules (β-tubulin) labeled with STAR635P in fixed U2OS cells. The pixel times of noisy input and ground-truth were 0.072 μs and 2.3 μs, respectively. We can clearly see significant improvement in SNR by comparing the predicted images to the noisy STED data (Fig. 1b), indicating our approach can reduce the pixel time of STED microscopy by >32-fold. Compared to other networks or deconvolution, our approach yields improved accuracy of predictions in terms of multi-scale structural similarity index (MS-SSIM), peak SNR (PSNR) and normalized mean squared error (NMSE) (Fig. 1c). Importantly, our method maintains the lateral resolution of STED images assessed by line profile analysis (Fig. 1d). For example, for the ground-truth STED image we estimated a resolution of 57 ± 1 nm whereas the predicted result showed 59 ± 1 nm. We also validated our approach on various subcellular targets (Supplementary Fig. 4) and two-color samples (Supplementary Fig. 5). It is noteworthy that the fixed sample data was captured using the resonant scanner.

**Fig. 2:**
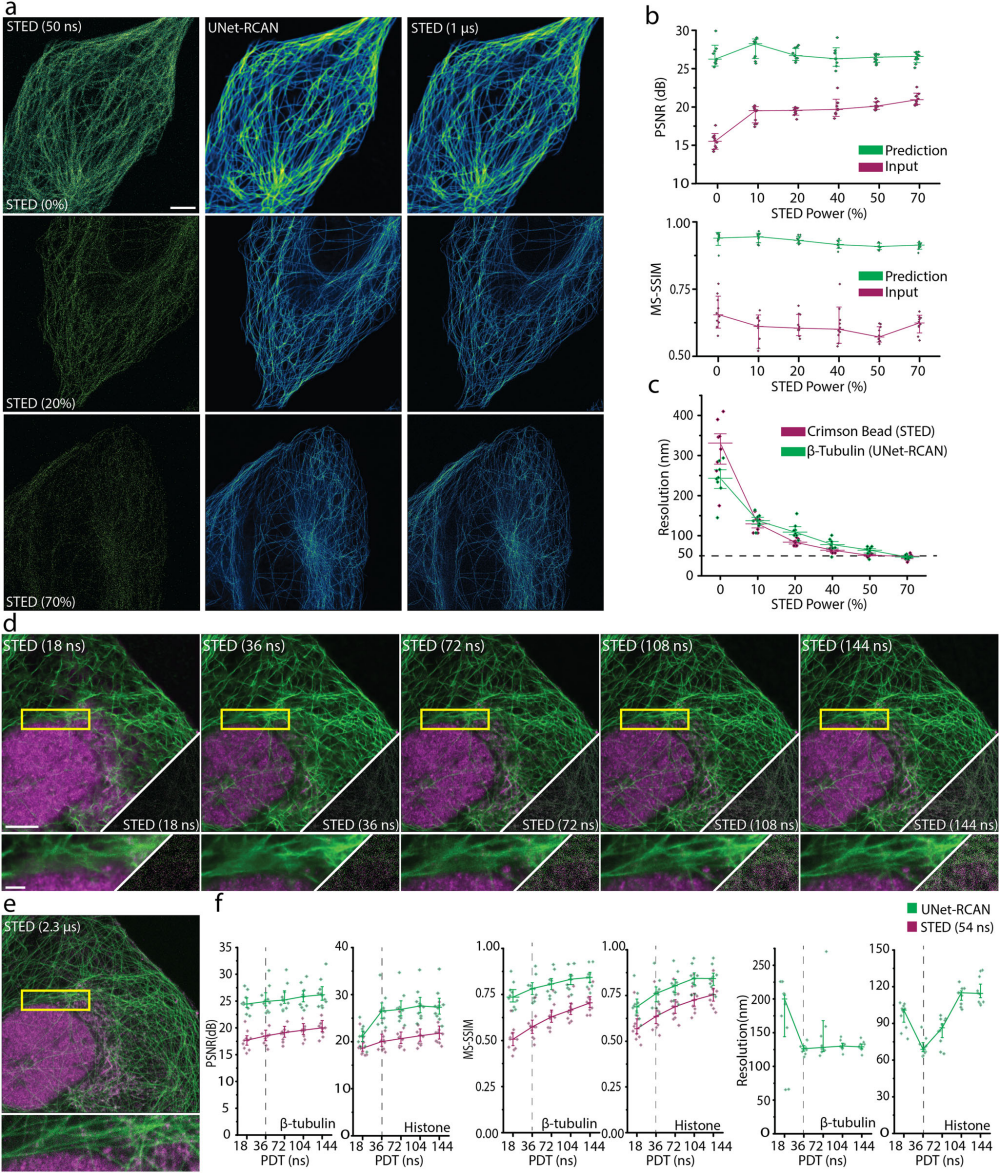
Dependence on STED power and SNR level. **a** Noisy (*Δt* = 50 ns), ground-truth (*Δt* = 1 μs) and UNet-RCAN STED images on β-tubulin (STAR635P) in U2OS cells. Six different datasets were generated by STED imaging with 0, 10, 20, 40, 50, and 70% of the STED power. **b** MS-SSIM and PSNR analysis for denoising results at each STED power by reference to ground-truth STED images (GT; *Δt* = 1 μs). Mean and standard deviation are displayed (*n* = 8). **c** The resolution of the prediction results (green) corresponds well with that of 20 nm crimson beads (magenta). The resolution was calculated by decorrelation analysis. The mean and standard error of the mean is displayed (*n* = 8). **d** Prediction results by UNet-RCAN of noisy two-color STED images of β-tubulin (STAR580, green) and histone (Atto647N, magenta) with pixel times of 18, 36, 72, 104, and 144 ns. **e** Ground-truth STED image with a pixel time of 2.3 µs. **f** PSNR, MS-SSIM, and resolution analysis for each pixel dwell time. Mean and standard deviation are displayed (*n* = 10). Scale bars, 5 µm and 1 µm (magnified regions).

We found that our method is robust at different STED powers (Fig. 2a–c). The spatial resolution of the predicted results is consistent with the scaling law of STED microscopy^21^. Although the SNR of STED images depends on numerous factors, including the excitation intensity, fluorophores, labeling density, pixel time, etc., we can estimate how reliable our prediction is given a certain level of SNR (Fig. 2d–f). We want to emphasize that our two-step deep learning approach has merit over others. Unlike content-aware image restoration (CARE)^14,22^, UNet-RCAN maintains the high spatial resolution of the STED images (Fig. 1d, e and Supplementary Fig. 6). Compared to cross-modality image restoration^17,18^ and deconvolution, our approach generates fewer artifacts in prediction, especially for low SNR images (Fig. 1f–h, Supplementary Fig. 7 and Supplementary Note 1). Nevertheless, one needs to beware of potential pitfalls of our approach. Like other deep learning methods, ours can produce deviations from the ground-truth as depicted in error maps in Fig. 1b. It is also unavoidable that the image quality parameters inherently drop for very low SNR images. Pixel based uncertainty metrics^14^ can provide the reliability of our results (Supplementary Fig. 8).

**Fig. 3:**
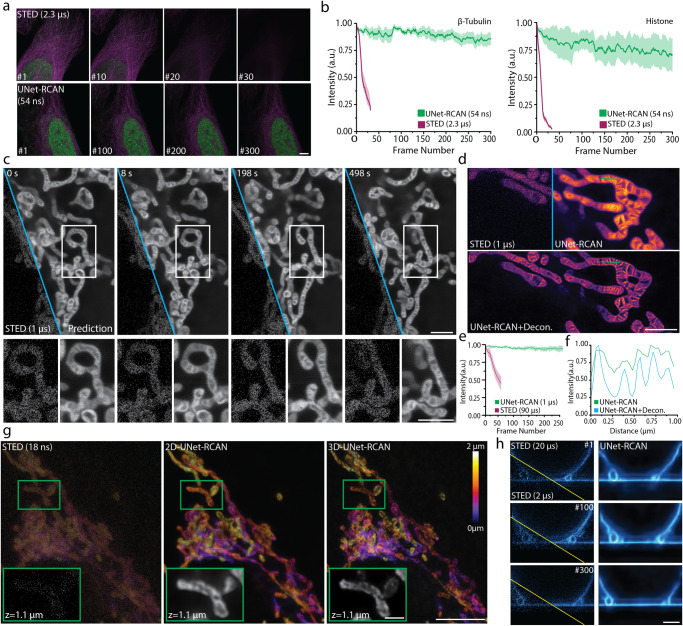
Reduced photobleaching and photodamage of STED imaging by UNet-RCAN. **a** Time-lapse STED images obtained by conventional STED (top, *Δt* = 2.3 µs) and UNet-RCAN using fast STED data (bottom, *Δt* = 0.054 µs). β-tubulin (STAR635P, magenta) and histone (Alexa 594, green) were imaged in U2OS cells. **b** Photobleaching analysis of two-color STED images used in **a**. Shaded areas are standard deviations of fluorescence intensities (*n* = 5). **c** Live-cell STED recording of mitochondrial cristae in HeLa cells (*Δt* = 1 μs) labeled with PK Mito Orange. **d** Denoising and deconvolved STED recording of mitochondrial cristae in COS-7 cells (*Δt* = 1 μs) labeled with PK Mito Orange **e** Fluorescence time traces of mitochondrial cristae in HeLa cells over 250 consecutive frames (2 s/frame). The fluorescence time trace of STED recording with *Δt* = 90 µs is displayed for comparison. The error bars denote the standard deviation. **f** Line profiles along a dashed line in **d**. **g** 2D- and 3D-UNet-RCAN prediction results for a noisy z-stack of 3D-STED imaging of TOM20 (Atto647N). The z pixel size is 65 nm. **h** Denoising results for *xz* time-lapse STED recording of membrane fusion dynamics (*Δt* = 2 μs) over 300 frames in comparison to ground-truth data (*Δt* = 20 μs). Scale bars, 5 μm **a**, **g**, 1 μm for the magnified regions **g**, and 2 μm **c**, **d**, **h**.

The low-exposure images used in our image restoration method are significantly less susceptible to photobleaching. While the signal level halved after 5–10 frames in conventional STED imaging of β-tubulin (STAR635P) and histone (Alexa 594), our approach maintained the signal for over 300 frames (Fig. 3a, b). Our approach also facilitates high-throughput STED imaging (Supplementary Fig. 9). It took 21 min to record 744 STED images (2048 × 2048 pixels) over a 1.0 × 0.78 mm^2^ region; otherwise, it would take ~14 h to do comparable imaging using traditional STED.

Next, we applied our image restoration approach to live-cell STED imaging. Its gentle illumination (*Δt* = 1 μs) enabled us to capture >200 frames of STED images of mitochondrial dynamics in HeLa cells with minimal phototoxicity, which is a ten-fold increase compared to the conventional STED (*Δt* = 90 μs) (Fig. 3c, e and Supplementary Videos 1–3). Our model preserved the shape of the original cristae images (Supplementary Figs. 10 and 11), and deconvolution can further improve their resolution (Fig. 3d, f and Supplementary Video 4).

It is straightforward to extend our approach to volumetric STED imaging. For this application, we trained a 3D UNet-RCAN using 3D stacks of STED images acquired by 2D or 3D STED (Fig. 3g, Supplementary Fig. 12). Our predicted results using fast 3D STED input images (*Δt* = 0.018 μs) clearly showed the hollow shape of mitochondria labeled to TOM20. The 3D model showed improvement in prediction accuracy compared to the 2D model, likely due to the effective consideration of noise^18^. The UNet-RCAN is also applicable to time-lapse 3D-STED *xz* imaging of fast dynamics. It revealed the fusion dynamics between a giant unilamellar vesicle and a supported bilayer^8^ with a temporal resolution of 315 ms/frame (*Δt* = 2 μs) compared to 3.15 s/frame (*Δt* = 20 μs) (Fig. 3h, Supplementary Video 5). It generally leads to noise-reduced data and even clearly recovering the membrane ghosts, a typical artifact for 3D STED images of membranes^23^.

In summary, restoring high SNR STED images is a powerful tool for fast, long-term super-resolution imaging. It is readily implementable without any hardware changes. When combined with other concepts like event-triggered imaging^24^ and/or single-photon avalanche diode (SPAD) array detector^25^, our method can further reduce the phototoxic effects of live-cell STED to a bare minimum. Similarly, our concept could be combined with an ultrafast scanning system^26^ to enable gentle live-cell nanoscopy at maximum speed.

## Methods

### Architecture of UNet-RCAN

We adopted a two-step prediction architecture from the multi-stage progressive image restoration (MPRNet)^27^, but it was modified for high-resolution fluorescence imaging as follows. The first subnetwork is a residual U-Net^19^, a convolutional neural network for image reconstruction through down-sampling and up-sampling operations like CARE^14^. An encoder consists of a residual convolution block—a first convolution layer, a leaky rectified linear unit (LeakyReLU; leakage factor = 0.3) as an activation function, and a second convolution layer, followed by a max-pooling (stride = 2) to extract the highlighted features (Fig. 1a and Supplementary Fig. 1). Each skip-connection in the residual blocks contains a convolution with a kernel size of 1 to refine the input before adding it to the output. A decoder consists of a transposed convolution, concatenation, and a residual convolution block to reconstruct the output image from the extracted features. To bypass the low-frequency information, we modified the architecture of residual U-Net by replacing the skip-connections between encoder and decoder paths with residual channel attention blocks (CAB; see below). We used three down-samplings and three up-samplings in the encoder and decoder paths, respectively. The initial number of convolutional filters is 64, which is doubled after each pooling in the encoder path while it is halved after each up-sampling in the decoder path. The output layer is a 1 × 1 convolution.

The second subnetwork is a residual channel attention network (RCAN)^20^, known to be a very deep convolutional neural network for super-resolution image reconstruction. Our RCAN network consists of 3 residual groups (RG) containing 8 CABs and a short skip-connection, a convolution layer, and a long skip connection. Each CAB consists of a convolution block with 64 channels, a global average pooling, a channel down-scaling convolution layer (filter size = 4), followed by a LeakyReLU and a channel upscaling convolution layer. Its output is passed through a sigmoid activation function and is used to rescale the input through multiplication. The upscaling module in the original RCAN was removed since the input and output in our network have the same shape. The number of residual groups and the filter size can increase to improve the performance at the cost of longer training time. All the convolution kernels have a size of 3 unless specified otherwise.

The input of the RCAN is the output of U-Net concatenated with the original noisy input image. While the RCAN network enhances the resolution of the denoised output by U-Net, the original noisy input guides to prevent the loss of spatial information during training. For 3D UNet-RCAN, all the 2D kernels used for convolutions, poolings, and up-samplings were replaced with three-dimensional vectors.

### Preparation of training dataset

We obtained ~20 pairs of noisy and high SNR STED images (2048 × 2048 pixels) for each target, from which training-set patches were created. The size of our training set for 2D or 3D networks was 1,200 patches (256 × 256 pixels) or 900 patches (160 × 160 × 16 pixels), respectively. Image normalization was performed on the image stacks such that each patch was normalized to its maximum. To exclude patches containing less information from the training dataset, we calculated the L2 norm of each patch, normalized it to the maximum of the norms of the training dataset, and discarded patches with their normalized norm being smaller than a threshold (0.2–0.4).

### Registration of noisy and ground-truth images

An essential step before training an image restoration model is a *xy*-drift correction between noisy and high SNR STED images. This was realized by calculating the cross-correlation of each pair of noisy and high SNR images in the Fourier domain. The drift between images was obtained by the maximum of the cross-correlation. We implemented this algorithm in MATLAB and applied it to the dataset before training our network.

### Preparation of semi-synthetic training dataset

Since Poisson noise is dominant in fast STED imaging, a semi-synthetic dataset can be generated by adding noise to high SNR STED data to make it resemble noisy STED data. We first adjusted the intensity of a high SNR STED image by multiplying it with a coefficient λ. We generated a random Poisson number at each pixel by using the pixel value as a random variable such that synthetic noisy images were prepared. We compared a histogram of this image with that of a noisy STED image obtained by fast STED imaging with a certain pixel dwell time and found the value of λ, which minimized the mean squared error (MSE) between the histograms. We used the average value of λ by repeating this procedure 5 times. It is important to discard the first bin of histograms and normalize them to their maximum before calculating MSE. We used this approach for restoring fast 3D STED images (Fig. 3g) and live-cell mitochondrial dynamics (Fig. 3c, d).

### Training UNet-RCAN

We optimized a loss function which is a weighted summation of Charbonnier loss (*L*_*char*_) and edge loss (*L*_*edge*_)^27^. The Charbonnier loss and edge loss are defined as:

1 $${L}_{char}(y,\hat{y})=\sqrt{{\Vert y-\hat{y}\Vert }^{2}+{\varepsilon }^{2}}$$

2 $${L}_{edge}(y,\hat{y})=\sqrt{{\Vert \varDelta (y)-\varDelta (\hat{y})\Vert }^{2}+{\varepsilon }^{2}}$$

where y is the ground-truth image, ŷ is the predicted image, Δ is the Laplacian operator, and *ε* is a constant set to 10^−3^. The Laplacian operator was implemented as a convolution of an image with a Laplacian filter. Combining the two loss functions prevents the smoothing effect that usually happens when training with the MSE loss function and ensures the reconstruction of super-resolution images^27,28^. The total loss function for training UNet-RCAN is defined as:

3 $$L(y,\hat{y})={L}_{char}(y,\hat{y})+\alpha {L}_{edge}(y,\hat{y})$$

where α is the weight parameter which is empirically set to 0.05 (ref. ^29^).

We implemented our model using Keras^30^ with a Tensorflow backend^31^ in Python. We used an Adam optimizer with the default parameters to minimize our loss function. The initial learning rate was set to 1 × 10^−4^, which is scheduled to change using the cosine annealing method^32^. We chose this method to prevent the model from converging to a local minimum. The batch size for training was set to 1 to prevent our GPU memory (12GB) from filling. The models were trained for 200 epochs (2D) and 100 epochs (3D) on an NVIDIA GeForce RTX 3080 Ti graphics card. The training times for 2D and 3D models were approximately 8 h and 24 h, respectively (Supplementary Tables 1 and 2). Representative loss curves of training and validation are depicted in Supplementary Fig. 13.

For STED power dependence experiments, each UNet-RCAN network was trained for restoring images of β-tubulin labeled with STAR635P, which were captured at 0, 10, 20, 40, 50, and 70% of STED power. To verify that our prediction results follow the scaling law of STED microscopy^21^, we compared the resolution of our predicted results with that of 20 nm crimson beads (ThermoFisher) at different STED powers.

### Training other networks

We implemented CARE in Keras, according to https://github.com/CSBDeep/CSBDeep. The model was trained on 1200 patches with 256 × 256 pixels, a batch size of 16, and an initial learning rate of 4 × 10^−4^. 2D-RCAN^20^ was implemented using Keras with 5 residual groups (RG) and 10 channel attention blocks (CAB) within each RG. The RG filter shape was set to 64, and the CAB filter shape was set to 4. The model was trained on 1,200 patches with 256 × 256 pixels, a batch size of 1, and an initial learning rate of 1 × 10^−4^. We trained these models by optimizing the MSE loss function using an Adam optimizer. Pix2pix^33^ was implemented using Keras according to https://github.com/phillipi/pix2pix. The model was trained on 1200 patches with 256 × 256 pixels with a batch size of 1 and an initial learning rate of 5 × 10^−5^.

To compare our two-step prediction approach to one-step prediction by modified U-Net or RCAN as described earlier, each network was separately trained for restoring STED images of microtubules. The U-Net filter shape was chosen to be [32,64,128], and the RCAN filter shape was set to 32 with 3 residual groups and 8 channel attention blocks. The filter shape of channel attention blocks was set to 4.

### Quantitative assessment of prediction results

To evaluate the predicted results, a test set of 10 different images with a shape of 2048 × 2048 was analyzed by peak signal-to-noise ratio (PSNR), normalized mean squared error (NMSE), and multi-scale structural similarity index (MS-SSIM) using the built-in functions of TensorFlow. Spatial resolution was quantified by either line profile analysis or an ImageJ plug-in for decorrelation analysis^34^ (Radius min = 0, Radius max = 1, Nr = 50, Ng = 10). Line profile analysis was performed by measuring the intensity profiles in 10 different regions of each image. A 2D Gaussian function was fitted to each line profile using Origin 2021b to measure the full width at half maximum (FWHM). The average and standard deviation of these parameters for all the predictions results are calculated and displayed in Supplementary Fig. 4 and Supplementary Tables 3–5. It is noteworthy to mention that the testing data was not included in the training process.

### STED microscopes

Confocal and STED images were acquired using a Leica SP8 3X STED with an oil objective (HC PL APO 100x/NA1.4, Leica) or an Abberior STED Expert Line with an oil objective (UPLXAPO 100x/NA1.45, Olympus). The depletion beams were pulsed lasers emitting at 775 nm. For the Leica system, the excitation power was set to 20%, and the images were detected with HyD detectors (a gain value of 20). We used a resonant scanning mode with a line speed of 8 kHz. The gating window was set to 0.4–12 ns. For 3D STED imaging, the z-STED was activated with 50% of the STED power. 3 line-averaging (*Δt* = 0.054 μs) or 128 line-averaging (*Δt* = 2.3 μs) was applied for collecting the noisy or the ground-truth data. For high-throughput imaging, an *xy* grid of 31 × 24 STED images with 20% overlap between tiles was obtained with 3-line-averaging and the Leica autofocusing system. For the Abberior system, the excitation power was set to 4.5%, and the images were detected with avalanche photodiodes. The gating window was set to 0.75–8 ns. We used a quad galvo scanner with a pixel time of 1 μs. Live-cell STED imaging was performed at room temperature. For details on the imaging conditions, please see Supplementary Table 6.

### Restoration of high SNR live-cell STED imaging on mitochondrial dynamics

We generated a semi-synthetic dataset as described above (Supplementary Figs. 10 and 11). Briefly, we used high SNR STED images (*Δt* = 90 μs) as ground-truth and generated noisy inputs which have comparable SNR to fast live-cell STED images of mitochondria (*Δt* = 1 μs). The trained network was applied to the noisy live-cell videos to restore high SNR STED time-lapse images.

### Photobleaching assessment

To compare the photobleaching effects of conventional STED and fast STED imaging with deep learning, five different field-of-views were imaged for each imaging modality. Image restoration was performed by UNet-RCAN on the fast STED data. To obtain the photobleaching curves, the L2 norm of the noisy data was calculated over the frames and normalized to the maximum of norms. This vector was applied to the prediction results normalized to their maximum over the frames. The average intensity of each frame for denoised fast STED and conventional STED images was plotted as a function of frame number.

### Cell culture

For imaging immunolabeled samples, U2OS cells (human bone osteosarcoma, HTB-96, ATCC) were grown in McCoy’s 5 A medium (ATCC) supplemented with 10% fetal bovine serum (FBS, Sigma-Aldrich, F2442) and 1% penicillin-streptomycin (ThermoFisher), and seeded on coverslips 2-3 days before experiments. For imaging mitochondria dynamics, HeLa^35^ or COS-7 cells were grown in Dulbecco’s Modified Eagle Medium (DMEM) with glutaMAX and 4.5 g/L glucose (ThermoFisher), 1% (v/v) penicillin-streptomycin (Sigma-Aldrich), 1 mM sodium pyruvate (Sigma-Aldrich), and 10% (v/v) FBS (Merck Millipore) at 37 °C in a 5% CO_2_ incubator. The cells were seeded in glass-bottom dishes (ibidi GmbH) one day prior to imaging.

### Immunofluorescence labeling

U2OS cells were fixed with 4% paraformaldehyde (Electron Microscopy Sciences, 15710) and 0.2% glutaraldehyde (Electron Microscopy Sciences, 16019) in phosphate buffered saline (PBS) for 15 min at room temperature, then washed in PBS. After incubation in 0.1% (w/v) sodium borohydride (Sigma-Aldrich) for 10 minutes, the cells were washed with PBS three times, followed by blocking with 3% bovine serum albumin (BSA, ThermoFisher) in PBS and permeabilization with 0.5% Triton-X 100 (Sigma-Aldrich) in PBS. When labeling microtubules, the cells were fixed with 0.6% paraformaldehyde, 0.1% glutaraldehyde, and 0.25% Triton-X 100 in PBS for 1 min at 37 °C. The cells were incubated in a primary antibody solution diluted to a final concentration of 2.5 µg/mL in PBS overnight at 4 °C. After washing three times in PBS, the cells were incubated in a secondary antibody solution diluted to a final concentration of 5 µg/mL in PBS overnight at 4 °C. After washing three times in PBS, a cover slip was mounted on a glass microscope slide using Mowiol (Sigma-Aldrich). Immunolabeling reagents are listed in Supplementary Table 7.

### Labeling in living cells

For one-color imaging, HeLa or COS-7 cells were stained with DMEM containing 250 nM PK Mito Orange (Confocal.nl)^36^ for 40 min, followed by three washing steps in DMEM. The cells were kept in the incubator for 1 h to remove unbound dyes. The culture medium was replaced with HEPES buffered DMEM containing 4.5 g/L glucose, L-glutamine, and 25 mM HEPES (ThermoFisher). For two-color imaging, COS-7 cells were transfected with Halo-KDEL^37^ using the JetPRIME transfection reagent (Polyplus) according to the manufacturer’s protocol. The next day, the cells were stained with DMEM supplemented with 250 nM PK Mito Orange and 500 nM 647-SiR-CA^38^ for 40 min at 37 °C. The cells were imaged at room temperature using the Abberior system.

### Preparation of membrane system for imaging vesicle dynamics

Giant unilamellar vesicles made of 1-palmitoyl-2-oleoyl-glycero-3-phosphocholine (POPC) and cholesterol (2:1 molar ratio) were prepared following the electroformation method^8^. A lipid mixture (5 µL, 1 g/L) dissolved in chloroform were spread onto platinum wires mounted in a custom made polytetrafluoroethylene chamber. The lipid mixture was dried with a gentle stream of N_2_ and subsequently submerged in a 300 mM sucrose buffer. The wires were connected to a function generator. A 10 Hz 2.0 V sine wave was applied for 1 h, with the frequency being reduced to 2 Hz for an extra 30 minutes. Supported lipid bilayers made of 1,2-dioleoyl-sn-glycero-3-phosphocholine (DOPC), 1,2-dioleoyl-snglycero-3-phosphoethanolamine (DOPE), and 1,2-dioleoyl-sn-glycero-3phospho-L-serine (DOPS) (molar ratio 4:3:3) were prepared following the spin coating method. The lipid mixture (25 µL of 1 g/L) dissolved in chloroform:methanol (1:1 volume ratio) were spin-coated (30 s, 3000 rpm) on plasma treated coverslips (#1.5). The coverslips were then mounted on AttoFluor chambers (ThermoFisher), hydrated in HEPES-buffered saline, and cleaned 10 times. The giant vesicles were then transferred to the supported lipid bilayer chamber and after labeling with 200 nM of the exchangeable membrane dye NR4A^8^ and let for 15 min to settle. To promote membrane fusion 10 mM CaCl_2_ dissolved in HEPES-buffered saline was added.

### Membrane dynamics imaging

Images were acquired on an Abberior Expert Line system^8^ equipped with a UPlanSApo 60 × /1.2 water immersion objective lens. Depletion in the *z* direction strongly depended on the correct adjustment of the objective lens correction collar. NR4A was excited with a 561 nm laser with a 10 µW laser power at the sample plane. Depletion was achieved using a 775 nm (40 MHz) with a power of 300 mW at the sample plane.

### Statistics and reproducibility

The network was trained and tested multiple times for the restoration of immunostained data to find the optimal set of hyperparameters. The number of training datasets was chosen by the quality of prediction results. Replicates were defined as images obtained from different field-of-views. For fixed samples, data was collected with two different STED microscopes and on different visits to assure the reproducibility of our model. Live-cell mitochondrial imaging was performed on two different cell lines.

### Reporting summary

Further information on research design is available in the Nature Portfolio Reporting Summary linked to this article.

### Supplementary information

Supplementary Information

Description of Additional Supplementary Files

Supplementary Data 1

Supplementary Video 1

Supplementary Video 2

Supplementary Video 3

Supplementary Video 4

Supplementary Video 5

Reporting Summary

## Data Availability

Data may be obtained from the authors upon reasonable request. The source data behind the graphs in the paper can be found in Supplementary Data 1.

## Code availability

The codes, sample data, and instruction guide are available at the GitHub repository (https://github.com/vebrahimi1990/UNet_RCAN_Denoising.git).
