# Author Correction: Deep learning enables fast, gentle STED microscopy

**DOI:** 10.1038/s42003-023-05222-1

**Published:** 2023-08-10

**Authors:** Vahid Ebrahimi, Till Stephan, Jiah Kim, Pablo Carravilla, Christian Eggeling, Stefan Jakobs, Kyu Young Han

**Affiliations:** 1 CREOL, The College of Optics and Photonics, University of Central Florida, Orlando, FL USA; 2 Department of NanoBiophotonics, Max Planck Institute for Multidisciplinary Sciences, Göttingen, Germany; 3 Department of Neurology, University Medical Center Göttingen, Göttingen, Germany; 4 Department of Cell and Developmental Biology, University of Illinois at Urbana-Champaign, Urbana, IL USA; 5 Leibniz Institute of Photonic Technology e.V., Jena, Germany, member of the Leibniz Centre for Photonics in Infection Research (LPI), Jena, Germany; 6 Faculty of Physics and Astronomy, Institute of Applied Optics and Biophysics, Friedrich Schiller University Jena, Jena, Germany; 7 Jena School for Microbial Communication, Friedrich Schiller University Jena, Jena, Germany; 8 Medical Research Council Human Immunology Unit, Weatherall Institute of Molecular Medicine, University of Oxford, Oxford, UK; 9 Translational Neuroinflammation and Automated Microscopy, Fraunhofer Institute for Translational Medicine and Pharmacology ITMP, Göttingen, Germany

**Keywords:** Fluorescence imaging, Machine learning

Correction to: *Communications Biology* 10.1038/s42003-023-05054-z, published online 27 June 2023.

In this article the funding from the ‘Deutsche Forschungsgemeinschaft (German Research Foundation; under Germany’s Excellence Strategy – EXC 2051 – Project-ID 390713860; project number 316213987 – SFB 1278; Instrument funding MINFLUX Jena INST 275_405_1), the State of Thuringia (TMWWDG), and the Free State of Thuringia (TAB; AdvancedSTED / FGZ: 2018 FGI 0022; Advanced Flu-Spec / 2020 FGZ: FGI 0031), and support by the integration into the Leibniz Center for Photonics in Infection Research (LPI, part of the BMBF national roadmap for research infrastructures)’ was omitted. The original article has been corrected.

