# Supplementary material for: Deep learning enables fast, gentle STED microscopy
Source: Commun Biol. 2023 Jun 27;6:674. doi: 10.1038/s42003-023-05054-z (PMC10300082; doi:10.1038/s42003-023-05054-z)
Supplement: Supplementary file 1 — Supplementary Information [file 42003_2023_5054_MOESM1_ESM.pdf]

Supplementary Information

Deep learning enables fast, gentle STED microscopy

Vahid Ebrahimi[1], Till Stephan[2,3], Jiah Kim[4], Pablo Carravilla[5,6], Christian Eggeling[5,6,7,8], Stefan Jakobs[2,3,9], Kyu Young Han[1,*]

[1]CREOL, The College of Optics and Photonics, University of Central Florida, Orlando, FL, USA

[2]Department of NanoBiophotonics, Max Planck Institute for Multidisciplinary Sciences, Göttingen, Germany

[3]Department of Neurology, University Medical Center Göttingen, Göttingen, Germany

[4]Department of Cell and Developmental Biology, University of Illinois at Urbana-Champaign, Urbana, IL, USA

[5]Leibniz Institute of Photonic Technology e.V., Jena, Germany, member of the Leibniz Centre for Photonics in Infection Research (LPI), Jena, Germany

[6]Faculty of Physics and Astronomy, Institute of Applied Optics and Biophysics, Friedrich Schiller University Jena, Jena, Germany

[7]Jena School for Microbial Communication, Friedrich Schiller University Jena, Jena, Germany

[8]Medical Research Council Human Immunology Unit, Weatherall Institute of Molecular Medicine, University of Oxford, Oxford, United Kingdom

[9]Translational Neuroinflammation and Automated Microscopy, Fraunhofer Institute for Translational Medicine and Pharmacology ITMP, Göttingen, Germany

*Correspondence to Kyu Young Han: kyhan@creol.ucf.edu

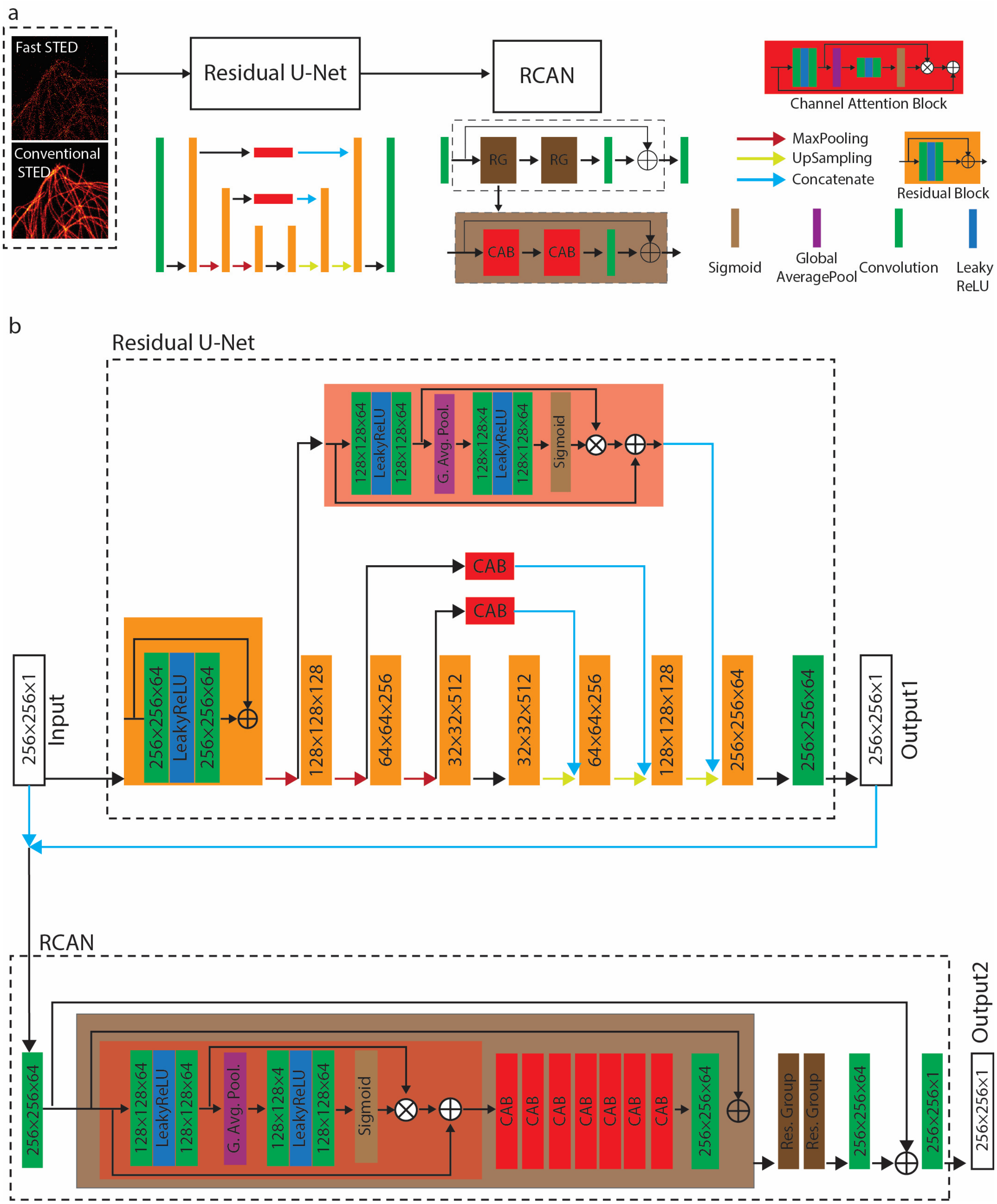

Supplementary Fig. 1, The architecture of UNet-RCAN for restoring fast STED imaging data. (a) The schematic of two-step prediction with UNet-RCAN. (b) Detailed architecture of UNet-RCAN. CAB, channel attention block; G. Avg. Pool., global average pooling; Res. Group, residual group. The model has one input and two outputs. Both output1 and output2 contribute to the loss function.

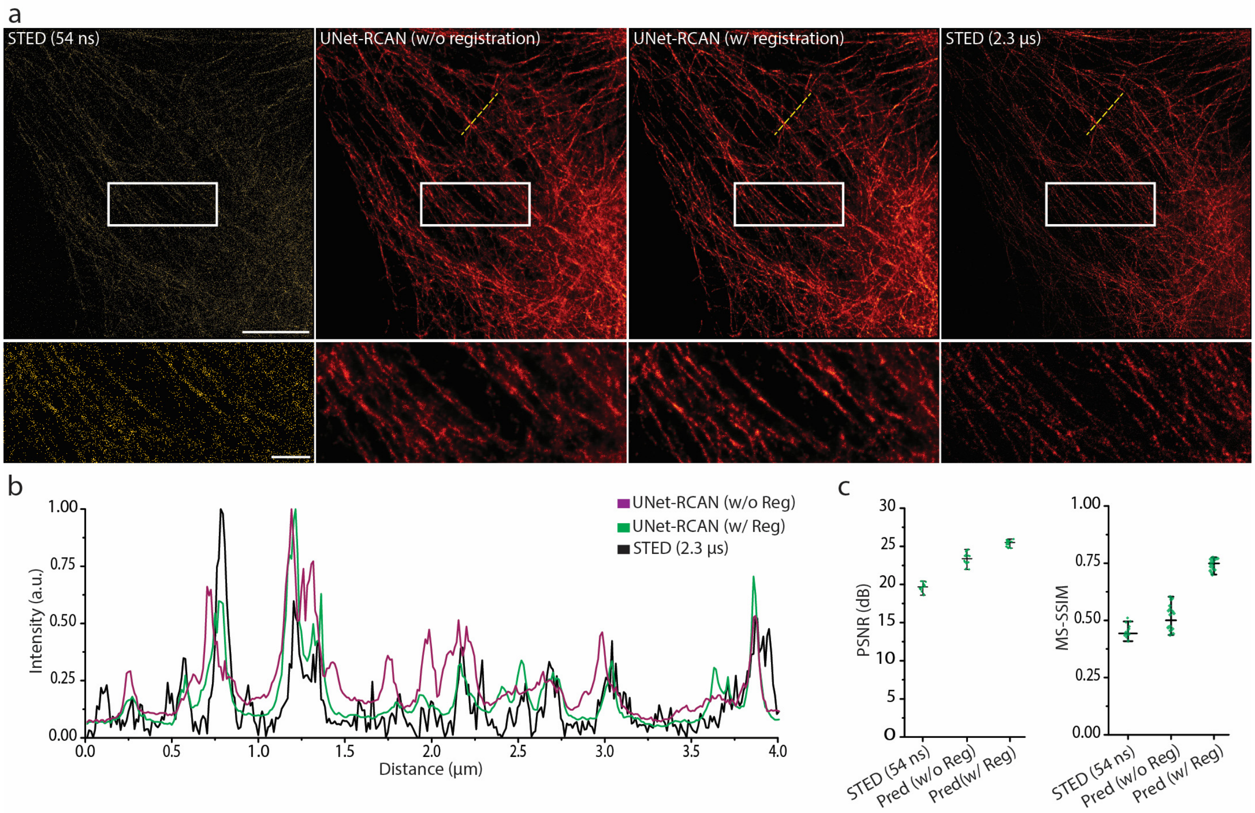

Supplementary Fig. 2, Registration of noisy input and ground-truth STED images. Registration improves prediction accuracy. Predicted STED images (a), line profiles (b), and quantitative assessment (c) by UNet-RCAN with and without registration of the noisy and ground-truth images by drift correction. Line profiles were measured along the yellow dashed lines. Scale bars, 5 µm and 1 µm (magnified regions).

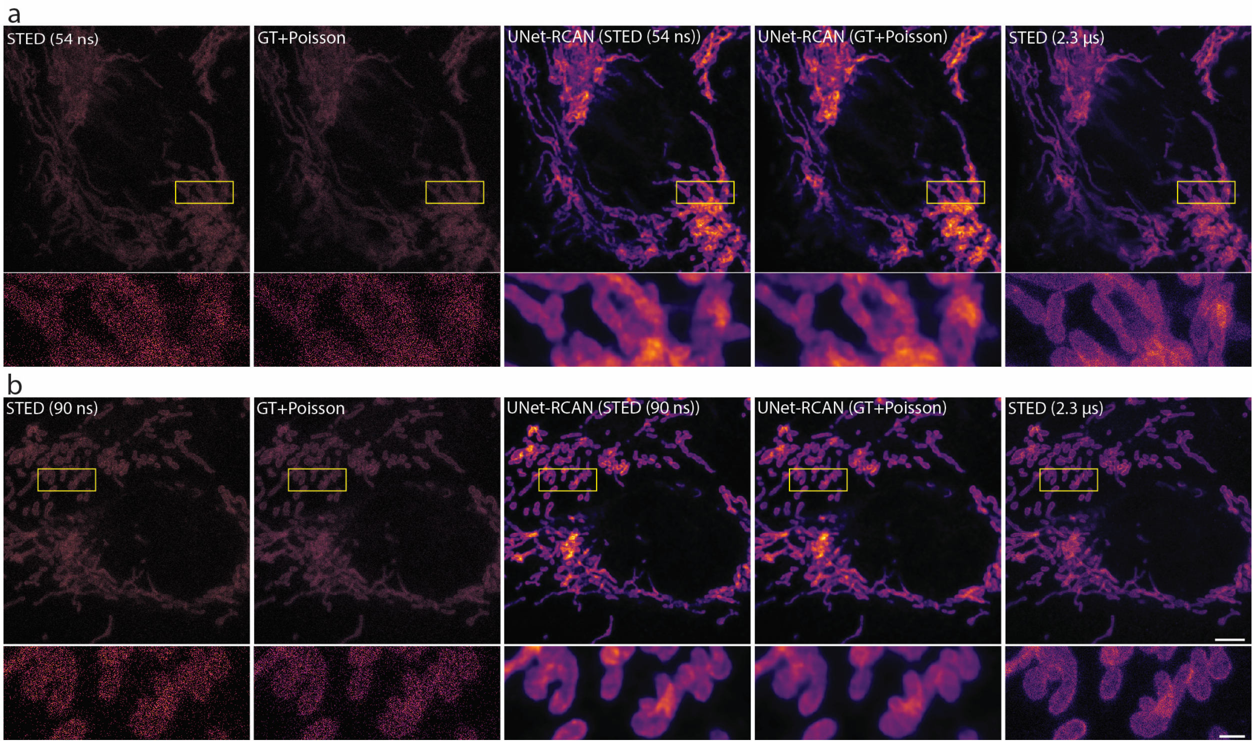

Supplementary Fig. 3, Comparison of semi-synthetic data and sequentially acquired noisy data. The predicted STED results from semi-synthetic data show similar results to those of well registered pairs of images. Different levels of SNR were tested, i.e., with a pixel time of 54 ns (a) or 90 ns (b) by adding Poisson noise to STED data with a pixel time of 2.3 µs. STED images of TOM20 labeled with Atto647N in U2OS cells. Scale bars, 5 µm and 1 µm (magnified regions).

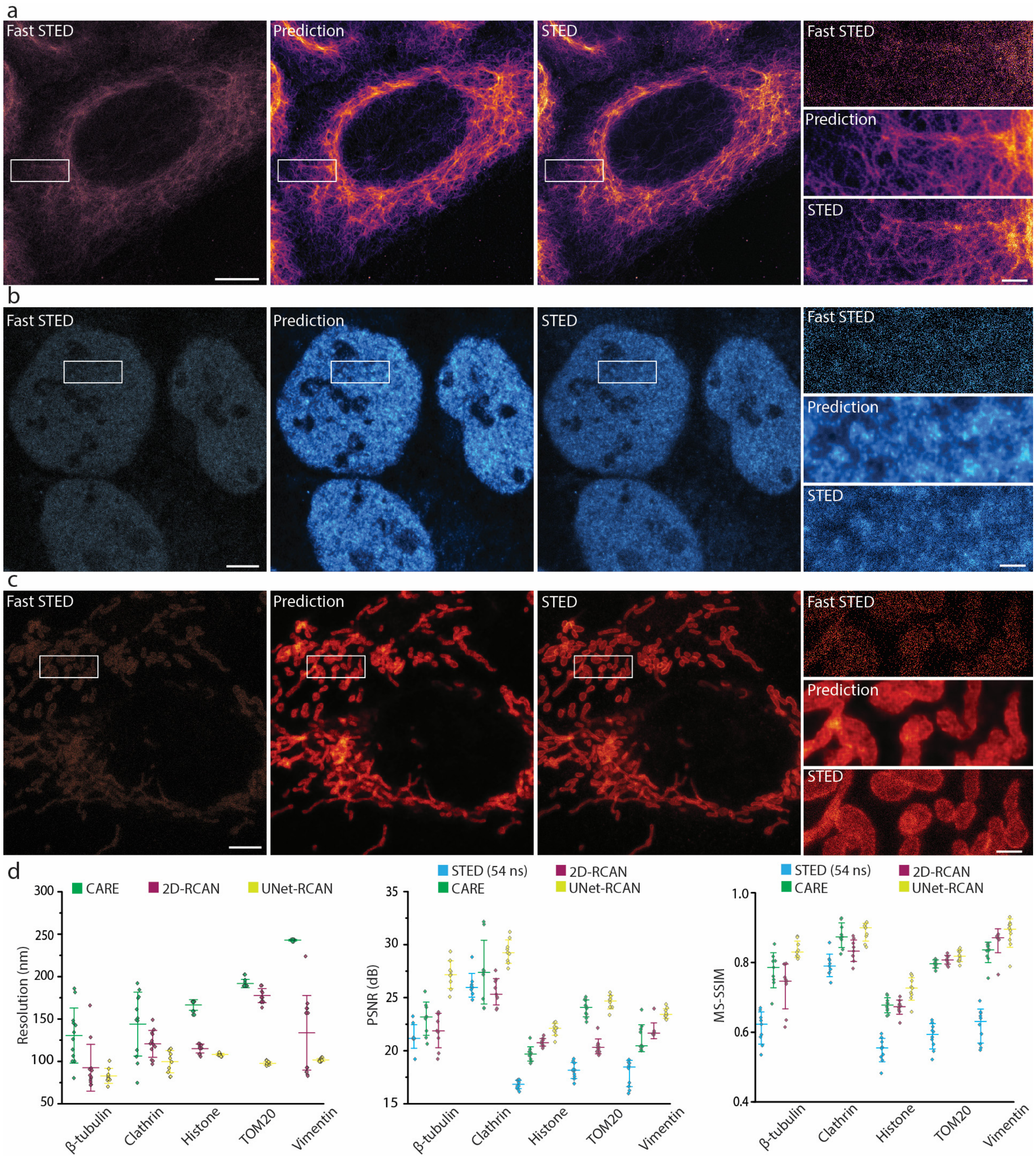

Supplementary Fig. 4, Image restoration results of 2D-STED images by UNet-RCAN on subcellular structures and comparison of the performances of UNet-RCAN, CARE, and 2D-RCAN. (a) Vimentin labeled with STAR635P, (b) Histone (H3K9ac) labeled with Atto647N, and (c) TOM20 labeled with Atto647N in fixed U2OS cells. Scale bars, 5 µm and 1 µm (magnified regions). (d) Five different markers were used for testing: β-tubulin (STAR635P), clathrin (STAR580), histone (Atto647N), TOM20 (Atto647N), and vimentin (STAR635P). PSNR and MS-SSIM were calculated with reference to ground-truth STED images (GT; Δt= 2.3 µs). The resolution was obtained by decorrelation analysis. Mean and standard deviation are displayed (n = 10).

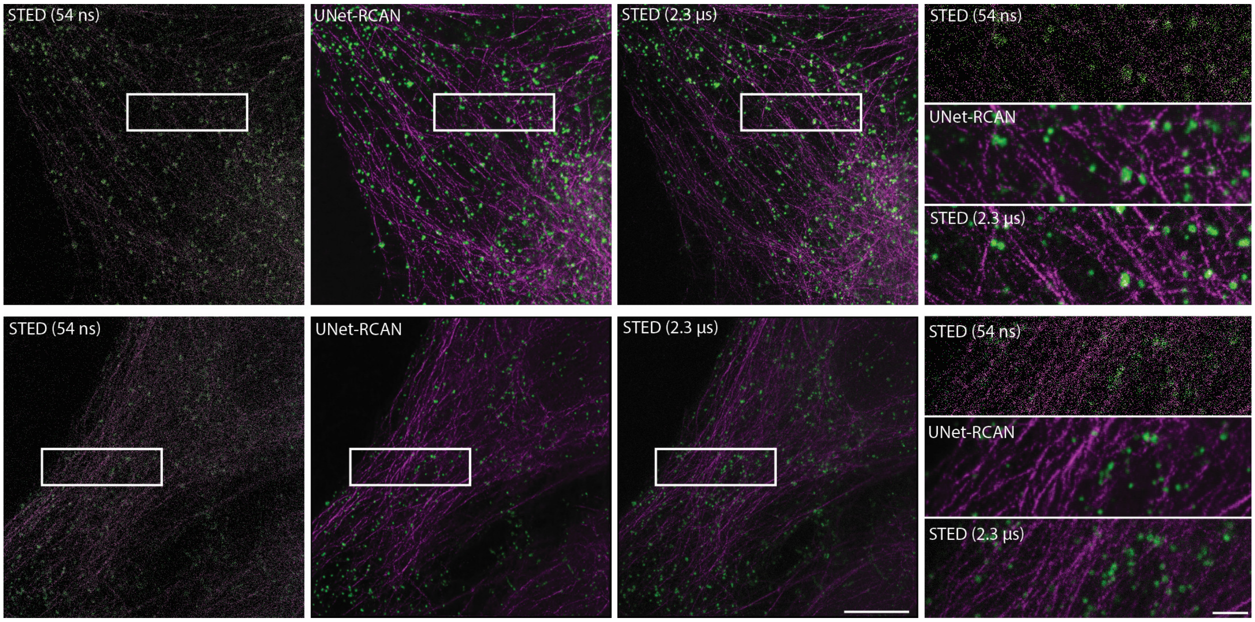

Supplementary Fig. 5, Restoring two-color fast STED imaging with UNet-RCAN. STED images of β-tubulin (STAR635P, magenta) and clathrin (STAR580, green) in U2OS cells with a pixel time of 54 ns. The ground-truth STED images were captured with a pixel time of 2.3 µs. Scale bar, 5 µm and 1 µm (magnified regions).

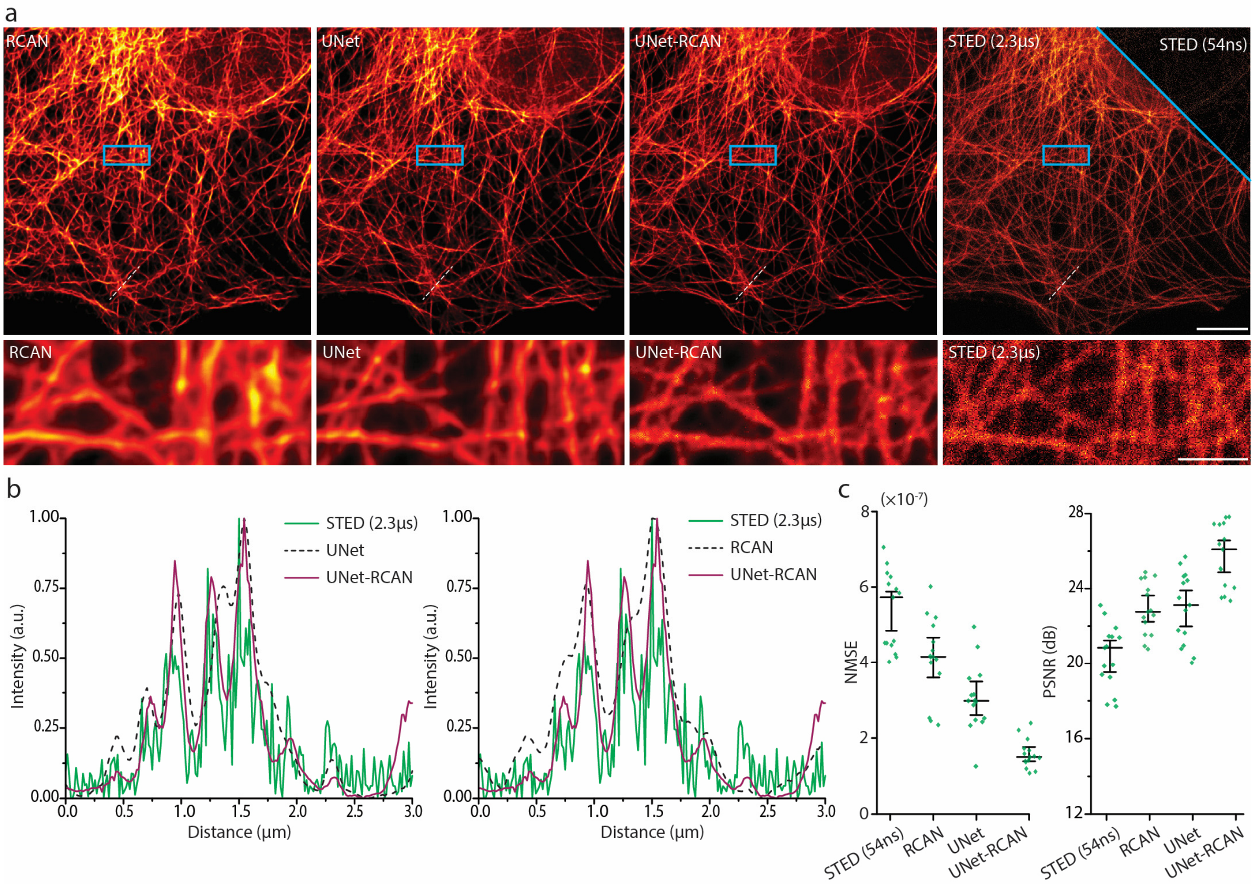

Supplementary Fig. 6, Comparison of the denoising performance of fast STED imaging with UNet-RCAN, residual U-Net, and 2D-RCAN. (a) β-tubulin labeled with STAR635P in fixed U2OS cells was imaged by a STED microscope with a dwell time of 54 ns (noisy input) and 2.3 µs (GT). The denoising is performed by UNet-RCAN, U-Net, and RCAN on the same noisy input. (b) Line profile measurements along the dashed line for GT and predictions by UNet-RCAN, U-Net, and RCAN. (c) Normalized mean squared error (NMSE) and PSNR of the predictions by UNet-RCAN, U-Net, and RCAN. The analysis shows the average and standard deviation for each parameter (n = 10). Scale bars, 5 µm and 1 µm for the magnified regions.

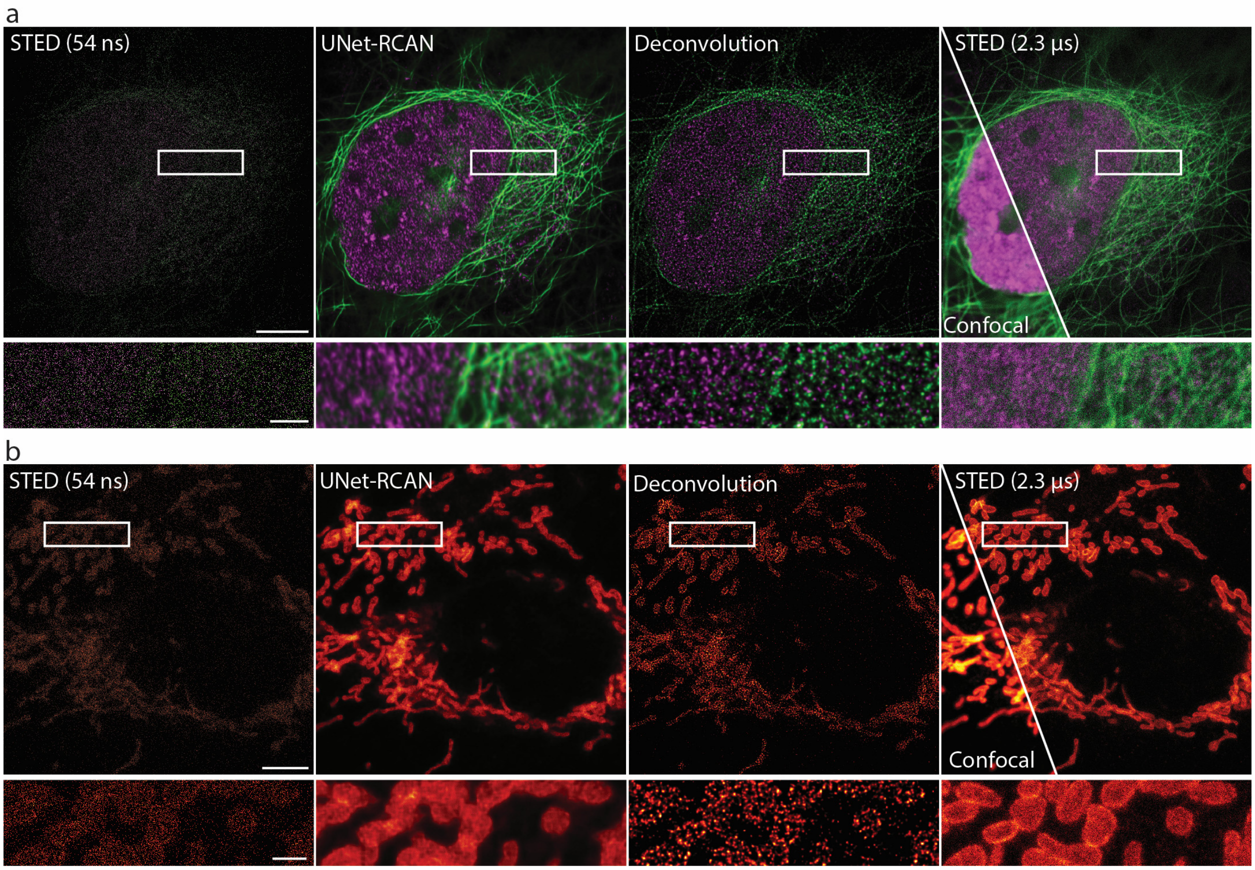

Supplementary Fig. 7, Comparison of denoising by UNet-RCAN and deconvolution. Denoising fast STED imaging of (a) β-tubulin (STAR580, green) and histone (Atto647N, magenta), and (b) TOM20 (Atto647N) in U2UOS cells. Separate denoising tasks were performed by UNet-RCAN and deconvolution on noisy STED data captured with a pixel time of 54 ns. Deconvolution was performed with Huygens software (See Methods). Scale bars, 5 µm and 1 µm (magnified regions).

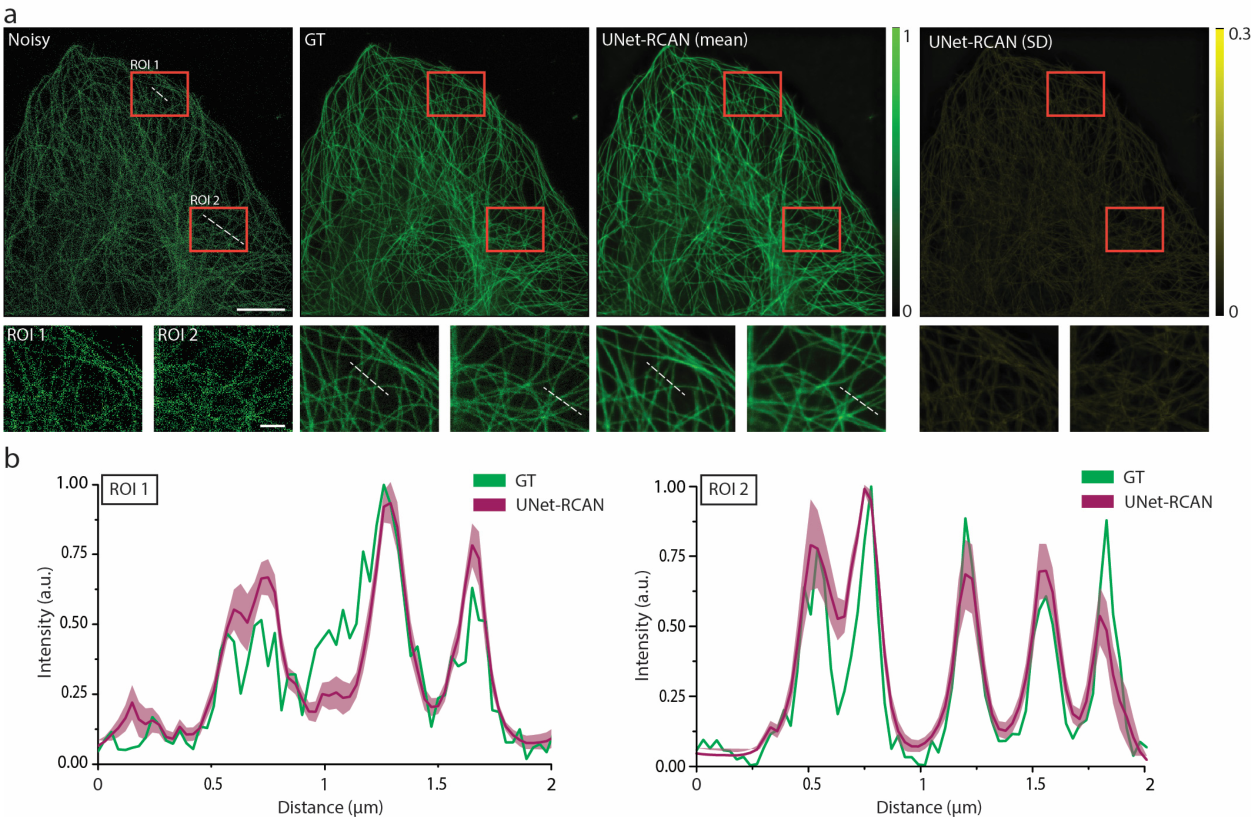

Supplementary Fig. 8, Prediction reliability by UNet-RCAN. (a) An ensemble is formed by training 10 UNet-RCAN networks to restore noisy STED microtubule data (STAR635P). An ensemble disagreement is measured by calculating the mean and standard deviation (SD) of the ensemble prediction results. (b) The mean and standard deviation of line profiles are measured in two regions (ROI 1 and ROI 2).

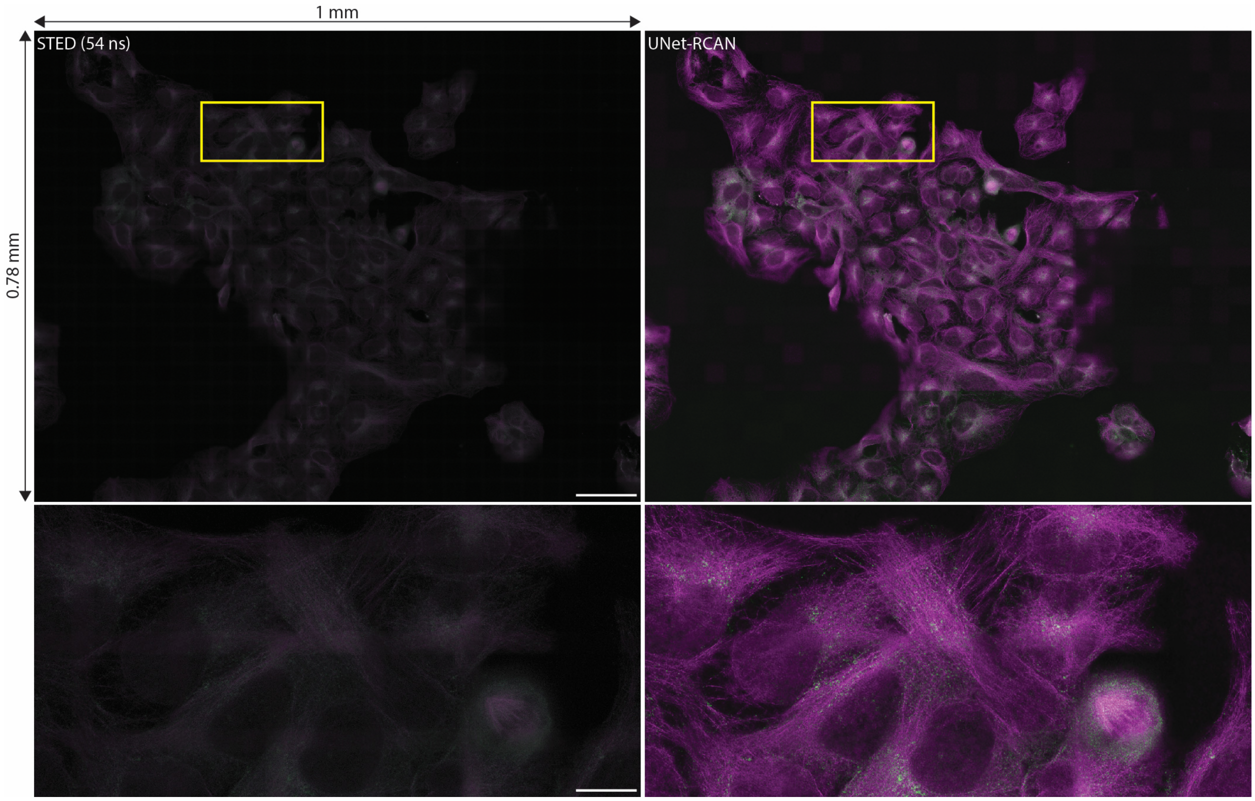

Supplementary Fig. 9, High-throughput STED imaging with UNet-RCAN. STED images of β-tubulin (STAR635P, magenta) and clathrin (STAR580, green) in U2OS cells with a pixel time of 54 ns. A grid of 31×24 images was denoised with UNet-RCAN and stitched together. Scale bars, 100 µm and 20 µm (magnified regions).

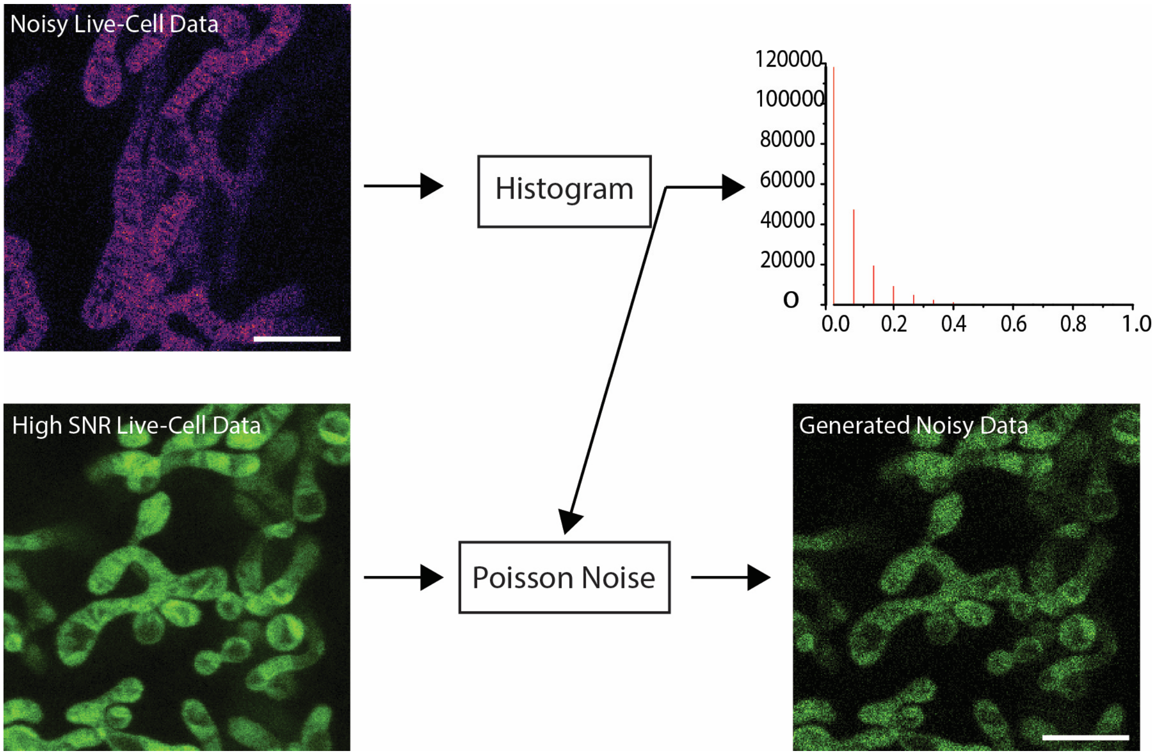

Supplementary Fig. 10, Semi-synthetic dataset generation. Poisson noise was applied to the high SNR STED images of cristae labeled with PK Mito Orange in HeLa cells to generate a pair of noisy and high SNR data for training UNet-RCAN. The amount of Poisson noise was adjusted such that the intensity histogram of the generated noisy data resembles that of the noisy live cell STED data (See Methods). Scale bars, 2µm.

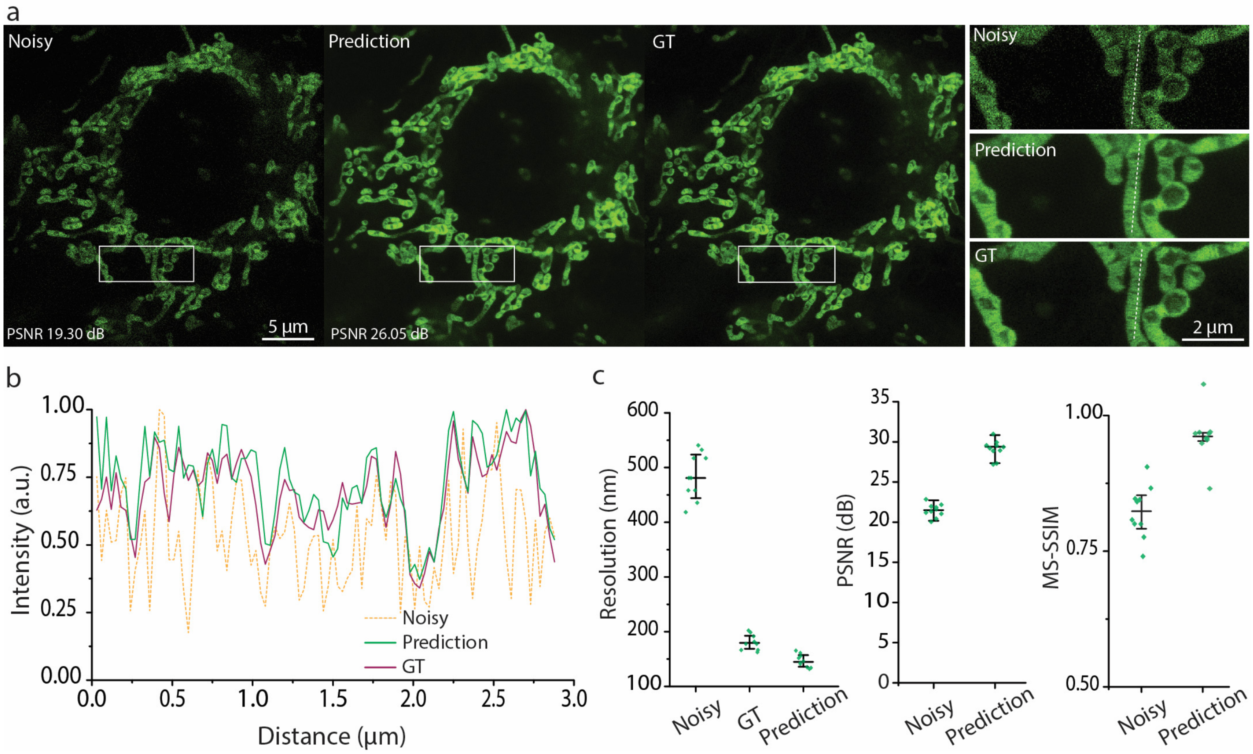

Supplementary Fig. 11, Denoising performance of UNet-RCAN on the semi-synthetic dataset. (a) Denoising results of cristae labeled with PK Mito Orange in HeLa cells. The GT data was captured with a dwelling time of 90 µs. The noisy data was generated by adding Poisson noise. The prediction is the denoising result by UNet-RCAN. (b) Line profiles of noisy, prediction, and GT data along the dashed lines in (a). (c) Resolution analysis by decorrelation, PSNR, and MS-SSIM calculations were performed on the prediction results by UNet-RCAN. Mean and standard deviation are displayed (n = 10).

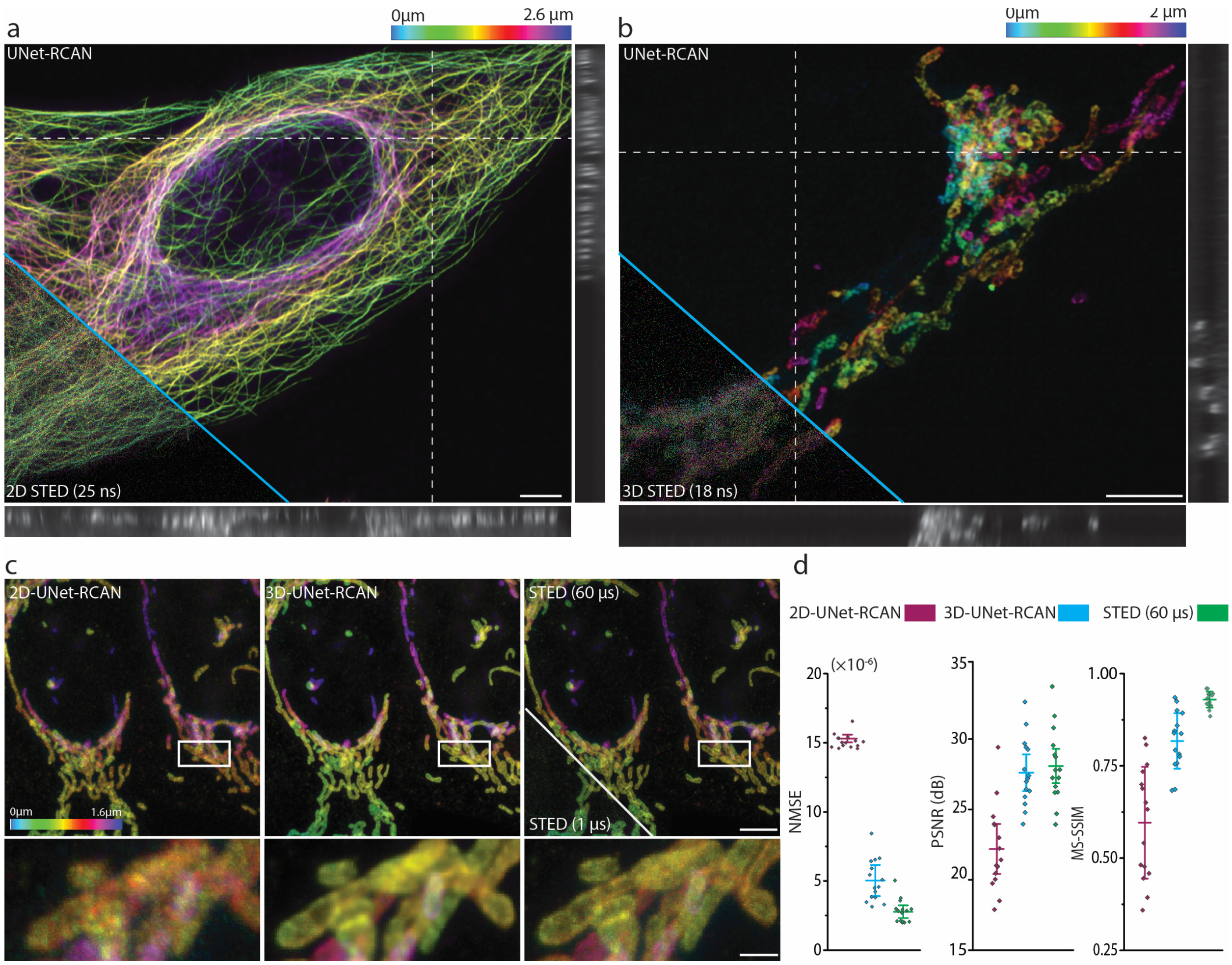

Supplementary Fig. 12, Prediction by 3D-UNet-RCAN on 2D and 3D STED imaging. 3D-UNet-RCAN prediction results for a noisy z-stack of (a) 2D-STED imaging of β-tubulin (STAR635P) and (b) 3D-STED imaging of TOM20 (Atto647N) in U2OS cells. The z pixel sizes are 170 nm and 65 nm, respectively. (c) Prediction results for a noisy z-stack of 3D STED imaging of TOM20 (Atto647N) by 2D- and 3D-UNet RCAN. The z pixel size is 40 nm. (d) NMSE, PSNR, and MS-SSIM analysis for prediction results by 2D- and 3D-UNet-RCAN (n=10). Scale bars, 5 μm and 1 μm for the magnified regions.

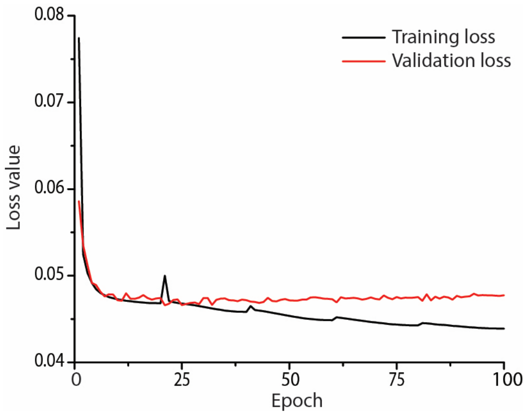

Supplementary Fig. 13, Training and validation loss curves for 2D-UNet-RCAN. The model was trained for denoising 2D STED imaging of microtubule (STAR635P) shown in Fig1. b.

Supplementary Note 1, Comparisons with other deep learning approaches

In cross-modality image restoration, a diffraction-limited confocal image is transformed into a super-resolved STED image with resolution enhancement by a deep convolutional neural network[1]. Different network architectures could perform this image transformation, such as generative adversarial networks (GAN)[2] or residual channel attention networks (RCAN)[3]. A transformation between confocal and STED imaging modalities at least requires a 3~5-fold resolution enhancement, i.e., from 250 nm to 50 nm; however, the cross-modality deep learning approaches have proven to be limited by a factor of 2-2.5 in terms of resolution enhancement[3]. Moreover, a lack of enough information often leads to exhibit artifacts.

Unlike the cross-modality image transformation, denoising is performed on noisy but super-resolved STED images to improve SNR. In denoising, the input data contains more information in terms of spatial resolution. This can help to reduce artifact generation and improve resolution enhancement. In Figs. 1f-h, we showed that denoising STED data clearly outperforms the cross-modality approach perceptually and according to the image quality assessment parameters.

Two popular network architectures suitable for denoising super-resolution data are UNet and RCAN. A UNet learns the features in an image dataset through convolutional layers and multiple down-sampling and up-sampling layers. Although the UNet effectively denoises diffraction-limited imaging data such as widefield images, its output does not reliably preserve high-frequency information. This is likely due to the fact that there is no mechanism to prioritize high-frequency information. Moreover, in UNet, a final image is reconstructed through downsampling and upsampling layers rather than applying the convolutional filters on the original noisy super-resolved data.

On the other hand, RCAN contains channel attention blocks and several skip connections, which help prioritize and maintain high-frequency information in super-resolution image reconstruction. Moreover, in RCAN, final super-resolved images are restored by applying filters on the original noisy data. This lowers the possibility of missing high-frequency information. However, our STED denoising results with RCAN show that although its final result is superior to UNet in terms of resolution, it generates more high-frequency artifacts, which may be due to its CAB building blocks, especially when the input SNR is extremely poor.

We showed that by combining UNet and RCAN, denoising could be effectively performed on fast STED data while we can maintain the super-resolution and prevent high-frequency artifact generation (Supplementary Fig. 6).

Supplementary Tables

Supplementary Table 1. Parameters and training time for CARE, 2D-RCAN, and UNet-RCAN.

	CARE	2D-RCAN	2D-UNet-RCAN	3D-UNet-RCAN
# Iterations per epoch	70	1,080	1,080	1080
Batch size	16	1	1	1
Patch size	256×256	256×256	256×256	160×160
Epochs	200	200	200	200
Number of parameters	3,790,850	3,944,073	16,684,270	152,935,406
Training time	1 h 17 m	11 h 40 m	8 h 10 m	30 h

Supplementary Table 2. Detailed computing hardware information.

Processor	AMD Ryzen 9 5900X 12-Core Processor 3.70 GHz
RAM	64 GB
GPU	NVIDIA GeForce RTX 3080 Ti

Supplementary Table 3. Performance comparison chart of UNet-RCAN, CARE, and 2D-RCAN in terms of SNR.

	Noisy	CARE	2D-RCAN	UNet-RCAN
β-tubulin	21.3±1.1 dB	23.0±1.6 dB	22.0±1.6 dB	27.2±1.3 dB
Clathrin	26.2±1.1 dB	27.4±3.0 dB	25.6±1.2 dB	29.3±1.1 dB
Histone	16.2±0.6 dB	20.4±0.8 dB	21.2±0.8 dB	21.6±0.9 dB
TOM20	18.0±0.7 dB	23.1±1.3 dB	20.9±0.8 dB	24.6±0.6 dB
Vimentin	17.7±0.9 dB	23.0±1.8 dB	23.0±1.1 dB	24.2±1.1 dB

Supplementary Table 4. Performance comparison chart of UNet-RCAN, CARE, and 2D-RCAN in terms of similarity.

	Noisy	CARE	2D-RCAN	UNet-RCAN
β-tubulin	0.61±0.04	0.77±0.05	0.73±0.06	0.83±0.02
Clathrin	0.79±0.03	0.87±0.03	0.83±0.03	0.88±0.02
Histone	0.53±0.06	0.67±0.04	0.66±0.04	0.70±0.06
TOM20	0.59±0.03	0.79±0.02	0.80±0.01	0.81±0.02
Vimentin	0.58±0.05	0.81±0.05	0.83±0.04	0.85±0.06

Supplementary Table 5. Performance comparison chart of UNet-RCAN, CARE, and 2D-RCAN in terms of resolution measured by decorrelation analysis.

	CARE	2D-RCAN	UNet-RCAN
β-tubulin	80±3 nm	71±3 nm	51±1 nm
Clathrin	122±5 nm	103±6 nm	81±4 nm
Histone	166±2 nm	115±2 nm	110±1 nm
TOM20	193±2 nm	179±3 nm	98±2 nm
Vimentin	243±1 nm	115±13 nm	101±1 nm

Supplementary Table 6. Acquisition settings of STED imaging.

<table>
<tr><th>Figures</th><th></th><th></th></tr>
<tr><td>1b</td><td rowspan="9">Exc. power = 20%
STED power = 50%
Resonant scanning
Gating: 0.4-12 ns
Leica STED</td><td>Fluorophore: STAR635P, λ_{exc} = 635 nm, λ_{STED} = 775 nm
Pixel time: 0.072 μs (noisy) and 2.3 μs (ground-truth)</td></tr>
<tr><td>3a
SF 2a,4a,5,6a,7a, 9</td><td>Fluorophore: STAR635P, λ_{exc} = 635 nm, λ_{STED} = 775 nm
Pixel time: 0.054 μs (noisy) and 2.3 μs (ground-truth)</td></tr>
<tr><td>3a</td><td>Fluorophore: Alexa 594, λ_{exc} = 594 nm, λ_{STED} = 775 nm
Pixel time: 0.054 μs (noisy) and 2.3 μs (ground-truth)</td></tr>
<tr><td>SF 12a</td><td>Fluorophore: STAR635P, λ_{exc} = 635 nm, λ_{STED} = 775 nm
Pixel time: 0.025 μs (noisy) and 1μs (ground-truth)</td></tr>
<tr><td>1f
SF 3a, 4b, 4c, 7a, 7b</td><td>Fluorophore: Atto647N, λ_{exc} = 647 nm, λ_{STED} = 775 nm
Pixel time: 0.054 μs (noisy) and 2.3 μs (ground-truth)</td></tr>
<tr><td>SF 3b</td><td>Fluorophore: Atto647N, λ_{exc} = 647 nm, λ_{STED} = 775 nm
Pixel time: 0.090 μs (noisy) and 2.3 μs (ground-truth)</td></tr>
<tr><td>SF 5, 7a, 9</td><td>Fluorophore: STAR580, λ_{exc} = 580 nm, λ_{STED} = 775 nm
Pixel time: 0.054 μs (noisy) and 2.3 μs (ground-truth)</td></tr>
<tr><td>2d, 2e</td><td>Fluorophore: Atto647N, λ_{exc} = 647 nm, λ_{STED} = 775 nm
Pixel time: [0.018,0.036,0.072,0.108,0.144] μs (noisy) and 2.3 μs (ground-truth)</td></tr>
<tr><td>2d, 2e</td><td>Fluorophore: STAR580, λ_{exc} = 580 nm, λ_{STED} = 775 nm
Pixel time: [0.018,0.036,0.072,0.108,0.144] μs (noisy) and 2.3 μs (ground-truth)</td></tr>
<tr><td>2a</td><td>Exc. power = 20%
STED power = [0%,10%,20%,50%,70%],
Resonant scanning
Gating: 0.4-12 ns
Leica STED</td><td>Fluorophore: STAR635P, λ_{exc} = 635 nm, λ_{STED} = 775 nm
Pixel time: 0.050 μs (noisy) and 1.0 μs (ground-truth)</td></tr>
<tr><td>3g
SF 10b, 12b</td><td>Exc. power = 20%
2D-STED power = 50%
z-STED power = 50%
Resonant scanning</td><td>Fluorophore: Atto647N, λ_{exc} = 635 nm. λ_{STED} = 775 nm
Pixel time: 0.018 μs (noisy) and 2.3 μs (ground-truth)</td></tr>
</table>

	Gating: 0.4-12 ns<br>Leica STED	
SM 1	Exc. power = 4.5%<br>STED power = 22%<br>Galvo scanning<br>Gating: 0.75-8 ns<br>Abberior STED	Fluorophore: PK Mito Orange, λ_{exc} = 561 nm, λ_{STED} = 775 nm<br>Pixel time: 30 μs, Line accumulation: 3
3c, 3d<br>SF 11a<br>SM 2, 3, 4	Exc. power = 4.5%<br>STED power = 22%<br>Galvo scanning<br>Gating: 0.75-8 ns<br>Abberior STED	Fluorophore: PK Mito Orange, λ_{exc} = 561 nm, λ_{STED} = 775 nm<br>Pixel time: 1 μs (noisy)
SM 3	Exc. power = 1%<br>STED power = 25%<br>Galvo scanning<br>Gating: 0.75-8 ns<br>Abberior STED	Fluorophore: 647-SiR-CA, λ_{exc} = 640 nm, λ_{STED} = 775 nm<br>Pixel time: 1 μs (noisy)
SF 12c	Exc. power = 5%<br>2D-STED power = 50%<br>z-STED power = 50%<br>Galvo scanning<br>Gating: 0.4-12 ns<br>Abberior STED	Fluorophore: Atto647N, λ_{exc} = 635 nm. λ_{STED} = 775 nm<br>Pixel time: 1 μs (noisy) and 60 μs (ground-truth)
3h,<br>SM 5	Exc. power = 35%<br>2D-STED power = 0%<br>z-STED power = 100%<br>Galvo scanning<br>Gating: 0 ns<br>Abberior STED	Fluorophore: NR4A , λ_{exc} = 561 nm, λ_{STED} = 775 nm<br>Pixel time: 2 μs (noisy) and 20 μs (ground-truth)

Supplementary Table 7. Immunolabeling conditions.

Figures	Primary antibody	Secondary antibody	Fluorophore
1b, 2a, 3a<br>SI 2a, 5,<br>6a,8a, 9,12a	Monoclonal Anti-β-Tubulin antibody produced in mouse, Sigma-Aldrich, T5293	Fab Fragment Goat Anti-Mouse IgG1, Jackson ImmunoResearch, 115-007-185	Abberior STAR 635P
2d, 2e<br>SI 7a,	Monoclonal Anti-β-Tubulin antibody produced in mouse, Sigma-Aldrich, T5293	Fab Fragment Goat Anti-Mouse IgG1, Jackson ImmunoResearch, 115-007-185	Abberior STAR 580

SI 5, 9	Anti-Clathrin heavy chain antibody (ab21679)	Fab Fragment Goat Anti-Rabbit IgG, Jackson ImmunoResearch, 111-007-008	Abberior STAR 580
1f,2d,2e<br>SI 4b, 7a	Anti-acetyl-Histone H3 (Lys9) in rabbit, Sigma-Aldrich, 07-352	Rabbit IgG (H&L) Antibody ATTO 647N Conjugated Pre-Adsorbed, ROCKLAND, 611-156-122	Atto 647N
3a	Anti-acetyl-Histone H3 (Lys9) in rabbit, Sigma-Aldrich, 07-352	Alexa Fluor® 594 AffiniPure F(ab')$_2$ Fragment Goat Anti-Rabbit IgG (H+L)	Alexa Fluor 594
3g<br>SI 3a, 3b, 4c,7b, 12b,12c	Anti-TOMM20 antibody - Mitochondrial Marker, abcam, ab78547	Rabbit IgG (H&L) Antibody ATTO 647N Conjugated Pre-Adsorbed, ROCKLAND, 611-156-122	Atto 647N
SI 4a	Anti-Vimentin antibody, Mouse monoclonal (V6389-200UL)	Fab Fragment Goat Anti-Mouse IgG1, Jackson ImmunoResearch, 115-007-185	Abberior STAR 635P

References

1. Wang, H.D. et al. Deep learning enables cross-modality super-resolution in fluorescence microscopy. *Nat Methods* **16**, 103-110 (2019).
2. Ouyang, W., Aristov, A., Lelek, M., Hao, X. & Zimmer, C. Deep learning massively accelerates super-resolution localization microscopy. *Nat Biotechnol* **36**, 460-468 (2018).
3. Chen, J.J. et al. Three-dimensional residual channel attention networks denoise and sharpen fluorescence microscopy image volumes. *Nat Methods* **18**, 678-687 (2021).
