# Supplementary material for: Deep learning enables fast, gentle STED microscopy
Source: Commun Biol. 2023 Jun 27;6:674. doi: 10.1038/s42003-023-05054-z (PMC10300082; doi:10.1038/s42003-023-05054-z)
Supplement: Supplementary file 2 — Description of Additional Supplementary Files [file 42003_2023_5054_MOESM2_ESM.pdf]

Description of Additional Supplementary Files

File name: Supplementary Data 1
Description: The source data behind the graphs in the paper

File name: Supplementary Video 1
Description: Time-lapse STED imaging of mitochondria dynamics with a pixel time of 90 µs. HeLa cells were labeled with PK Mito Orange.

File name: Supplementary Video 2
Description: Fast deep-learning STED imaging of mitochondria dynamics with a pixel time of 1 µs. HeLa cells were labeled with PK Mito Orange.

File name: Supplementary Video 3
Description: Two-color live-cell deep-learning STED imaging of mitochondria (green) and ER (magenta) in HeLa cells with a pixel time of 1 µs. Mitochondria was labeled with PK Mito Orange, and ER was labeled with SiR-Halo.

File name: Supplementary Video 4
Description: Deep-learning live-cell STED imaging with deconvolution. COS-7 cells were labeled with PK Mito Orange.

File name: Supplementary Video 5
Description: Denoising fast 3D STED xz imaging of giant unilamellar vesicles (GUV) labeled with NR4A with a pixel time of 2 µs
