# Supplementary material for: Deep learning enables fast, gentle STED microscopy
Source: Commun Biol. 2023 Jun 27;6:674. doi: 10.1038/s42003-023-05054-z (PMC10300082; doi:10.1038/s42003-023-05054-z)
Supplement: Supplementary file 9 — Reporting Summary [file 42003_2023_5054_MOESM9_ESM.pdf]

nature portfolio

Corresponding author(s): Kyu Young Han

Last updated by author(s): Jun 10, 2023

Reporting Summary

Nature Portfolio wishes to improve the reproducibility of the work that we publish. This form provides structure for consistency and transparency in reporting. For further information on Nature Portfolio policies, see our Editorial Policies and the Editorial Policy Checklist.

Statistics

For all statistical analyses, confirm that the following items are present in the figure legend, table legend, main text, or Methods section.

n/a	Confirmed	
☐	☒	The exact sample size (*n*) for each experimental group/condition, given as a discrete number and unit of measurement
☐	☒	A statement on whether measurements were taken from distinct samples or whether the same sample was measured repeatedly
☒	☐	The statistical test(s) used AND whether they are one- or two-sided<br>*Only common tests should be described solely by name; describe more complex techniques in the Methods section.*
☒	☐	A description of all covariates tested
☒	☐	A description of any assumptions or corrections, such as tests of normality and adjustment for multiple comparisons
☐	☒	A full description of the statistical parameters including central tendency (e.g. means) or other basic estimates (e.g. regression coefficient) AND variation (e.g. standard deviation) or associated estimates of uncertainty (e.g. confidence intervals)
☒	☐	For null hypothesis testing, the test statistic (e.g. *F*, *t*, *r*) with confidence intervals, effect sizes, degrees of freedom and *P* value noted<br>*Give P values as exact values whenever suitable.*
☒	☐	For Bayesian analysis, information on the choice of priors and Markov chain Monte Carlo settings
☒	☐	For hierarchical and complex designs, identification of the appropriate level for tests and full reporting of outcomes
☒	☐	Estimates of effect sizes (e.g. Cohen's *d*, Pearson's *r*), indicating how they were calculated

Our web collection on statistics for biologists contains articles on many of the points above.

Software and code

Policy information about availability of computer code

Data collection	For image denoising on fixed samples, the images were collected by Leica SP8 3X STED. For denoising on live cell samples, the images were collected by Abberior STED Expert Line.
Data analysis	UNet-RCAN was used for denoising and cross-modality image transformation. The code is available from GitHub (https://github.com/vebrahimi1990/UNet_RCAN_Denoising.git).<br>Resolution calculation by image decorrelation analysis was performed by ImageJ (ImageDecorrelationAnalysis_plugin).<br>Deconvolution was performed by Huygens Essential (22.04).

For manuscripts utilizing custom algorithms or software that are central to the research but not yet described in published literature, software must be made available to editors and reviewers. We strongly encourage code deposition in a community repository (e.g. GitHub). See the Nature Portfolio guidelines for submitting code & software for further information.

Data

Policy information about availability of data

All manuscripts must include a data availability statement. This statement should provide the following information, where applicable:

- Accession codes, unique identifiers, or web links for publicly available datasets
- A description of any restrictions on data availability
- For clinical datasets or third party data, please ensure that the statement adheres to our policy

Data may be obtained from the authors upon reasonable request.

Human research participants

Policy information about studies involving human research participants and Sex and Gender in Research.

Reporting on sex and gender	n/a
Population characteristics	n/a
Recruitment	n/a
Ethics oversight	n/a

Note that full information on the approval of the study protocol must also be provided in the manuscript.

Field-specific reporting

Please select the one below that is the best fit for your research. If you are not sure, read the appropriate sections before making your selection.

☒ Life sciences ☐ Behavioural & social sciences ☐ Ecological, evolutionary & environmental sciences

For a reference copy of the document with all sections, see nature.com/documents/nr-reporting-summary-flat.pdf

Life sciences study design

All studies must disclose on these points even when the disclosure is negative.

Sample size	The exact sample size can be found in the Main Text and Supplementary Information.
Data exclusions	For training the model, we excluded the patches that did not contain sufficient amount of information. The details can be found in Method section (Preparation of training dataset). For testing, no data was excluded.
Replication	All the codes, data, and protocols are available for replicating the results of our experiments. All the STED denoising models were tested on at least 10 different testing data. The denoising results were quantified by averaging at least 10 different testing data.
Randomization	No allocation in experimental groups were performed.
Blinding	All the deep learning models were blindly tested with the data that was not used for training.

Reporting for specific materials, systems and methods

We require information from authors about some types of materials, experimental systems and methods used in many studies. Here, indicate whether each material, system or method listed is relevant to your study. If you are not sure if a list item applies to your research, read the appropriate section before selecting a response.

Materials & experimental systems

n/a	Involved in the study	
☐	☒	Antibodies
☐	☒	Eukaryotic cell lines
☒	☐	Palaeontology and archaeology
☒	☐	Animals and other organisms
☒	☐	Clinical data
☒	☐	Dual use research of concern

Methods

n/a	Involved in the study	
☒	☐	ChIP-seq
☒	☐	Flow cytometry
☒	☐	MRI-based neuroimaging

Antibodies

Antibodies used	mouse anti-β-tubulin, Sigma-Aldrich (T5293), anti-clathrin heavy chain (ab21679), rabbit anti-acetyl-histone H3 (Lys9), Sigma-Aldrich (07-352), anti-TOMM20 antibody - mitochondrial marker, abcam (ab78547), mouse anti-vimentin antibody, (V6389-200UL), fab goat anti-mouse IgG1, Jackson ImmunoResearch (115-007-185), fab goat anti-rabbit IgG, Jackson ImmunoResearch (111-007-008), rabbit IgG (H&L) ATTO 647N conjugated pre-adsorbed, ROCKLAND (611-156-122), alexa fluor 594 AffiniPure $F(ab')_2$ goat anti-rabbit IgG (H +L).
Validation	All the antibodies have been extensively used in previous studies. We compared the staining patterns with images in other literatures.

Eukaryotic cell lines

Policy information about cell lines and Sex and Gender in Research

Cell line source(s)	The following cell lines were used:<br>U2OS (ATCC), HeLa, COS-7
Authentication	The used cell lines were not authenticated.
Mycoplasma contamination	Cell lines were not tested for mycoplasma contamination. However, we often examined DAPI images to check potential contamination.
Commonly misidentified lines<br>(See ICLAC register)	The used cell lines were not commonly misidentified.
